# Construction of a risk map to understand the vulnerability of various types of cancer patients to COVID‐19 infection

**DOI:** 10.1002/mco2.53

**Published:** 2021-01-21

**Authors:** Jian He, Xufang Yang, Hui Wang

**Affiliations:** ^1^ State Key Laboratory of Oncogenes and Related Genes Center for Single‐Cell Omics School of Public Health Shanghai Jiao Tong University School of Medicine Shanghai China; ^2^ Department of Pathophysiology Mudanjiang Medical University Mudanjiang Heilongjiang China

**Keywords:** *ACE2*, cancer, COVID‐19, prognosis, tumor‐infiltrating lymphocytes

## Abstract

COVID‐19 is leading to a global pandemic and invades human cells via *ACE2*. *ACE2* was found to be abundantly expressed in many organs and cells. However, there is no evidence about the potential risk of various types of cancer patients vulnerable to the infection of COVID‐19. To obtain a risk map that indicates the novel coronavirus vulnerability of different types of cancer, we analyzed in this work the RNA sequencing datasets of cancer patients. By interrogating the datasets, we not only identified the cancer types vulnerable to COVID‐19 attacks, but also we reported that variations in the mRNA expression level of *ACE2* correlate to various prognosis phenomenon in different types of cancer cohorts, and illustrated the underlying mechanism involved or may be related to lymphocytes infiltration. From these discoveries, we constructed an infection risk map, which indicates the vulnerability of different types of cancer to COVID‐19 infection, also elucidated the correlationship between *ACE2* and the prognosis of cancer. We found that high *ACE2* expression levels lead to high risk of COVID‐19 infection and poor prognosis of breast invasive carcinoma (BRCA), while better prognosis in ovarian serous cystadenocarcinoma (OV) patient cohorts. Moreover, our study demonstrated that this different pattern may correlate with the immune infiltration level.

## Graphical abstract

This infection risk map indicating the vulnerability of different types of cancer to COVID‐19 infection, high *ACE2* expression levels leading to higher risk of COVID‐19 infection and poorer prognosis of breast cancer, while better prognosis in ovarian cancer patients. The underlying mechanism is that this different pattern may correlate with immune infiltration level. This may provide potential clues for further investigating the pathogenesis of COVID‐19 infection of cancer patients.

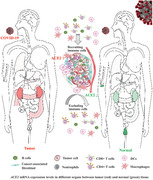

**Abbreviations**
BLCA: bladder urothelial carcinoma
BRCA: breast invasive carcinoma
CESC: cervical squamous cell carcinoma and endocervical adenocarcinoma
COAD: colon adenocarcinoma
ESCA: esophageal carcinoma
GBM: glioblastoma multiforme
HNSC: head and neck squamous cell carcinoma
KICH: kidney chromophobe
KIRC: kidney renal clear cell carcinoma
KIRP: kidney renal papillary cell carcinoma
LGG: brain lower grade glioma
LIHC: liver hepatocellular carcinoma
OV: ovarian serous cystadenocarcinoma
PAAD: pancreatic adenocarcinoma
PCPG: pheochromocytoma and paraganglioma
PRAD: prostate adenocarcinoma
READ: rectum adenocarcinoma
SARC: sarcoma
STAD: stomach adenocarcinoma
THCA: thyroid carcinoma
UCEC: uterine corpus endometrial carcinoma
UCS: uterine carcinosarcoma
UVM: uveal melanoma

## 1 INTRODUCTION

A novel human coronavirus‐induced pneumonia (World Health Organization [WHO] named COVID‐19 now) is rapidly spreading and caused a pandemic outbreak globally since December 2019.^1^ Besides the evidence of respiratory symptoms such as acute respiratory failure, fever, and cough, other clinical symptoms of SARS infection for example cardiovascular, urinary, digestive systems failure have been reported.^2^, ^3^, ^4^ The spike protein (S protein) of coronavirus helps the virus to enter target cells. This entry process relays on the binding of the surface unit (S1) of the S protein to the cell receptors, which could help the virus attach to the surface of target cells. Moreover, this procedure needs to trigger the S protein by cellular proteases, which requires the cleavage of the S protein at S1/S2 and S2' sites and allows the viral membrane and cell membrane to fuse, which is a procedure driven via S2 subunits.^5^ COVID‐19 was found with high similarity in sequence (∼80%) with SARS‐CoV, so this novel coronavirus is also named SARS‐CoV‐2, moreover it shares the identical receptor angiotensin‐converting enzyme (*ACE*) 2 with the SARS‐CoV.^5^ The interface of SARS‐S/*ACE2* has been clarified, and the efficiency of *ACE2* usage has been considered to be the crucial determinant of SARS‐CoV transmissibility.^6^ SARS‐S and SARS‐2‐S were found share ∼76% identity of amino acids and SARS‐2‐S also employs *ACE2* for host cell entry, which is like SARS‐S.^7^ So *ACE2* is considered to be the receptor in host cells for binding to this novel virus. Therefore, the target cells with the expression of *ACE2* are susceptible to COVID‐19 infection, for example AT2 cells in lung.^8^ So in the same way, any organ that expresses *ACE2* may be susceptible to COVID‐19 infection, and many research groups have reported this situation.^9^, ^10^, ^11^ All these findings indicate the potential risk of various cell types and organs with high‐expression *ACE2* will be vulnerable to COVID‐19 attacks.

The protein encoded by *ACE2* belongs to the ACE family of dipeptidyl carboxydipeptidases. This functional enzyme not only catalyzes the cleavage of angiotensin I into angiotensin 1‐angiotensin 9, but also could cleave angiotensin II into vasodilator angiotensin 1‐7.^12^ *ACE2* is the key enzyme in the renin‐angiotensin system (RAS), both of which are involved in pathological vessel growth and beneficial angiogenesis and it is also related to tumorigenesis.^12^, ^13^ Moreover, many researchers have demonstrated that the tumor‐infiltrating lymphocytes (TILs) play an important part in intervening the chemotherapy response and enhancing clinical prognosis especially survival rates of different cancer types,^14^, ^15^ such as tumor‐associated macrophages (TAMs)^16^, ^17^, ^18^ and tumor‐infiltrating neutrophils (TINs), and they also impact the outcomes of cancers.^19^, ^20^, ^21^, ^22^ Therefore, it is also essential to clarify the immunophenotyping of tumor immunocyte interactions and description of novel immunotherapy targets/strategy in cancer therapy.

Here, we determined the mRNA expression level of *ACE2* in different cancer patient cohorts. Also, we found that different degrees of *ACE2* level correlate to the prognosis in different types of cancer and the underlying mechanisms involved might be related to lymphocytes infiltration. Our study may provide an explicit evidence of the influence of COVID‐19 on high and low vulnerable cancer patients and the association between the expression level of *ACE2* and the prognosis of various cancer types, attracting more attention to survival rate evaluation of the cancer patients recovered from COVID‐19 infection.

## 2 RESULTS

### 2.1 The expression levels of *ACE2* in different human cancers

**FIGURE 1:**
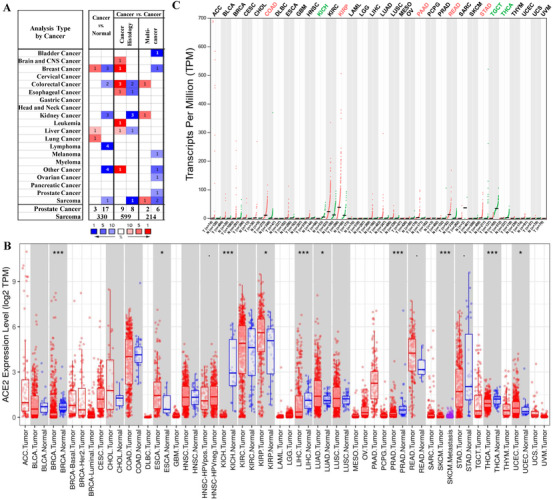
*ACE2* mRNA expression levels in different types of human cancers in different databases. A, Increased or decreased *ACE2* in datasets of different cancers compared with normal tissues in the Oncomine database. Cell color is determined by the best gene rank percentile for the analyses within the cell. B, Human *ACE2* expression levels in different tumor types from TCGA database. One category of cancer is in one box. *P‐*value significance codes: .*P* < .1, *P < .05, **P < .01, ***P < .001. C, *ACE2* expression profile across all tumor samples and paired normal tissues (dot plot). Each dot represents expression of samples. The gene expression profile across all tumor samples and paired normal tissues (bar plot). The height of bar represents the median expression of certain tumor type or normal tissue. Red means the expression in tumor tissues significantly higher than paired normal tissues and green means the expression in tumor tissues significantly lower than paired normal tissues.

To study the varied expression levels of *ACE2* in tumor and normal tissue of multiple cancer types, the *ACE2* mRNA expression levels were analyzed using the three main online databases (Oncomine, GEPIA2, EBI database). This investigation demonstrated that the mRNA expression level of *ACE2* was higher in breast, liver, and lung cancers, respectively, compared to the paired normal tissues (cancer *vs*. normal) (Figure 1A). Moreover, the *ACE2* was highly expressed in brain and CNS, breast, colorectal, esophageal, kidney, liver cancer, leukemia, and sarcoma in tumor tissues (cancer versus cancer) (Figure 1A). The details of the expression levels of *ACE2* in different types of cancers are categorized and summarized in Table S1.

In order to evaluate *ACE2* expression level in cancers, we determined the levels of *ACE2* expression employing the RNA‐seq datasets of multiple cancer types in the Cancer Genome Atlas (TCGA). The varied expression levels between tumor and adjacent normal tissues for *ACE2* across each type of TCGA tumor are demonstrated in Figure 1B. The expression level of *ACE2* was significantly higher in the tumor tissue of esophageal carcinoma (ESCA), kidney renal papillary cell carcinoma (KIRP), lung adenocarcinoma, rectum adenocarcinoma (READ), and uterine corpus endometrial carcinoma (UCEC), whereas lower in breast invasive carcinoma (BRCA), kidney chromophobe (KICH), liver hepatocellular carcinoma (LIHC), pancreatic adenocarcinoma (PAAD), prostate adenocarcinoma (PRAD), thyroid carcinoma (THCA), and stomach adenocarcinoma (STAD) compared with the adjacent normal tissue (Figure 1B). The dot plots generated by GEPIA are given profiling gene/isoform expression across multiple cancer types, with each dot demonstrating a specific tumor or normal sample. The differential mRNA expression level for *ACE2* between the tumor and matched TCGA normal and GTEx data across all TCGA tumors by GEPIA is exhibited in Figure 1C. The level of *ACE2* was aggregately expressed in colon adenocarcinoma (COAD), READ, PAAD, KIRP, and STAD, whereas it was lower in KICH, THCA, and testicular germ cell tumors compared to the adjacent normal GTEx tissue parts (Figure 1C).

**FIGURE 2:**
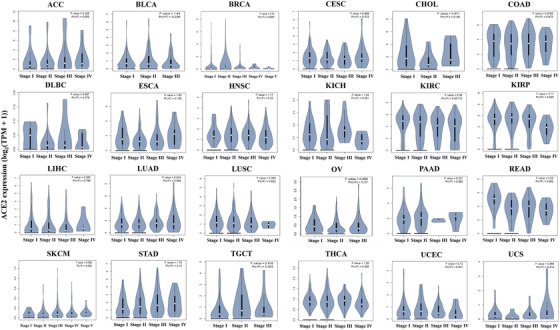
*ACE2* mRNA expression levels in different stages of different types of human cancers.

We also studied the *ACE2* expression in different stages of different types of cancers. We found that *ACE2* expression was higher in the earlier stage(s) of lymphoid neoplasm diffuse large B‐cell lymphoma, kidney renal clear cell carcinoma (KIRC), and READ, whereas higher in the later stage of STAD (Figure 2).

### 2.2 Prognostic potential of *ACE2* across various types of cancer

**FIGURE 3:**
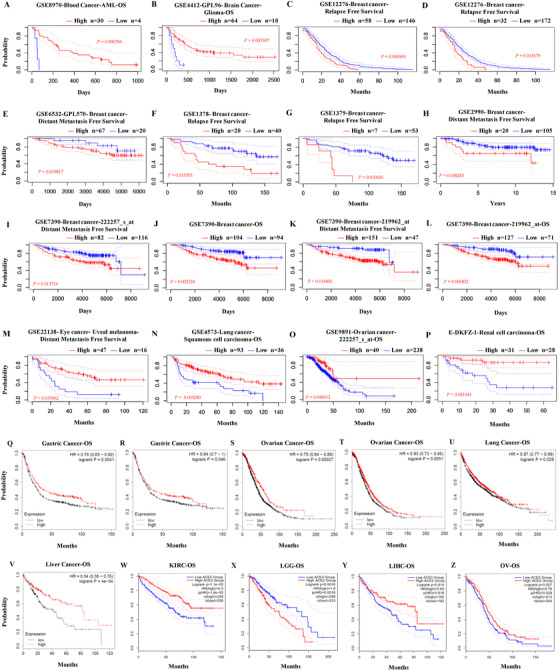
Kaplan‐Meier survival curves comparing the high and low expression of *ACE2* in different types of cancers in the PrognoScan (A‐P), Kaplan‐Meier plotter (Q‐V), and GIEPA (V‐Y) databases. A‐P, The better prognosis in cohorts of blood cancer, brain cancer, eye cancer, and lung cancer and poorer prognosis in six different cohorts of breast cancer were shown to correlate with higher *ACE2* expression in PrognoScan. Survival curves of OS in (A) one blood cancer cohort (GSE8970 [n = 34, *P =* .000394]), (B) one brain cancer cohort (GSE4412 [n = 74, *P =* .003507]), (J) one breast cancer cohort (GSE7390 [n = 198, *P =* .002524]), (C‐G) relapse‐free survival in different breast cancer cohorts (GSE12276, GSE1378, GSE1379), and (E,H,I,K) DMFS in four different breast cancer cohorts (GSE 6532‐GPL570, GSE 2990, GSE7390‐222257_s, GSE7390‐219962), (M) one eye cancer cohort (GSE22138 [n = 63, *P =* .033962]), and (N) one lung cancer cohort (GSE4573 [n = 129, *P =* .009280]); 95% confidence intervals for each group are also indicated by dotted lines. Q‐V, The better prognosis in two different cohorts of gastric cancer, two different cohorts of ovarian cancer, and one cohort each of lung cancer and liver cancer separately in KM plotter. W‐Z, *ACE2* expression significantly impacts prognosis in four types of cancers, including KIRC (n = 516), LGG (n = 481), LIHC (n = 364), and OV (n = 416) cohorts in GIEPA database, and shows better OS in KIRC, LIHC, and OV.

We determined whether the mRNA expression level of *ACE2* was associated with the prognosis across specific cancer patient cohorts. The effects of *ACE2* expression on the various survival rates were assessed by using the PrognoScan database. The detailed relationship between the expression level of *ACE2* and prognosis potential of various cancers are listed in Table S2. We found that *ACE2* expression level significantly impacts overall survival (OS) in breast cancer and ovarian cancer (Table S2 and Figure 3). A bunch of cohorts (GSE12276, GSE6532‐GPL570, GSE1378, GSE1379, and GSE7390) of breast cancer demonstrated that higher *ACE2* level was correlated to poor prognosis (relapse‐free survival [RFS] HR = 1.12, 95% CI = 1.02‐1.22, Cox *P* = .0156786; RFS/DMFS HR = 3.36, 95% CI = 1.21‐9.30, Cox *P* = .0198172; RFS HR = 1.38, 95% CI = 1.06‐1.79, Cox *P* = .0155929; RFS HR = 1.36, 95% CI = 1.08‐1.73, Cox *P =* .0100558; DMFS HR = 1.19, 95% CI = 1.04‐1.36, Cox *P* = .0137138; OS HR = 1.23, 95% CI = 1.08‐1.41, Cox *P* = .00252401) (Table S2 and Figure 3C‐L). So it is conceivable that high expression level of *ACE2* is an independent risk factor leading to a poorer prognosis in breast cancer patients, and hazard ratio demonstrated here indicates *ACE2* expression is not a protective factor in breast cancer. On the contrary, different ovarian cancer cohorts (GSE9891, 229962, 222257) in three databases showed that high expression level of *ACE2* was associated with better prognosis (OS HR = 0.63, 95% CI = 0.40‐1.00, Cox *P* = .0485116; OS HR = 0.75, 95% CI = 0.64‐0.88, Cox *P* = .00027; OS HR = 0.83, 95% CI = 0.73‐0.95, Cox *P* = .0051; OS HR (high) = 0.65, Cox *P* = .016) (Table S2 and Figure 3O,S,U,Y). Also, it is conceivable that high expression level of *ACE2* is an independent risk factor leading to a better prognosis in ovarian cancer patients, and hazard ratio which was demonstrated here indicates *ACE2* expression is a protective factor in ovarian cancer. Also, high *ACE2* expression significantly impacts RFS and DMFS in breast cancer cohorts (Figure 3), and leads to better prognosis in some other types of cancer (Figure 3).

To further analyze the prognostic characteristics of *ACE2* gene in different types of cancer, we employed Kaplan‐Meier plotter database to access the *ACE2* prognostic value. Similarly, a better prognosis pattern in ovarian cancer was shown to correlate with higher *ACE2* expression (Figure 3O,S,U).

In addition to using Kaplan‐Meier and PrognoScan plotter databases, TCGA database was also employed to determine the prognostic characteristics of *ACE2* in different types of cancers via GEPIA2. We assessed the relationships between the level of ACE2 and prognostic potential in 33 types of cancer. *ACE2* expression significantly affects prognosis in four types of cancers, including KIRC, brain lower grade glioma (LGG), LIHC, and ovarian serous cystadenocarcinoma (OV) (Figure S1). High *ACE2* expression levels were associated with better prognosis potential of OS in KIRC, LIHC, and OV, but negative influence in LGG.

These results demonstrated that the prognostic potential of *ACE2* in some types of cancers and the differential *ACE2* expression could lead to different prognostic values depending on the type of cancers.

### 2.3 The expression level of *ACE2* impacts the ovarian cancer prognosis in different stages and treatments

In this part, we studied the association with the expression level of *ACE2* and different clinical characteristics in order to better understand the relevance and mechanisms of the expression level of *ACE2* in cancers, especially in different clinical stages of breast cancer and ovarian cancer patients.

High expression level of *ACE2* was in relation to better OS of serous subtype rather than endometrioid subtype and better PFS in the endometrioid subtype of the ovarian cancer patients, respectively. High *ACE2* expression level was also associated to better OS in the early stage of the progress of ovarian cancer (Table S3). Point of interest was that higher level of *ACE2* was in correlation to better OS in the higher grade (2 + 3 and 3) than in lower grade (1, 1 + 2, and 2) but was not associated with PFS. High *ACE2* expression level was correlated with poorer OS in ovarian cancer patient cohorts without TP53 mutation (Table S3).

The results above imply that the level of *ACE2* could affect the prognosis potential in ovarian cancer patients of the early cancer staging but not in relation to OS and PFS of late stages. Also, we discovered that *ACE2* expression level was not associated with OS in any subtype or status in OV (ER, PR, HER2 status, lymph node status, intrinsic subtype, TP53 status, and the pietenpol subtype) and neither in any grade in breast cancer (Tables S3 and S4). This interesting phenomenon combined with the different survival rate patterns in Figure 3 indicate that the correlation of *ACE2* expression and the prognosis of different cancers depend on the different mechanisms in tumorigenesis and development.

Nowadays, the main treatments of ovarian cancer are debulking and chemotherapy; in this project, we found that higher level of *ACE2* was associated with better OS in both debulking groups (Table S5). Different OS patterns were demonstrated in chemotherapy treatments. Higher *ACE2* level was associated with better prognosis potential in the groups who underwent platin, taxol, taxol + platin, and paclitaxel chemotherapy treatments (Table S5). But this phenomenon does not appear in breast cancer (Table S6).

### 2.4 *ACE2* is associated with immune infiltration level in BRCA and OV

TILs are considered independent predictors of survival rates, especially OS, in various types of cancers.^23^, ^24^, ^25^ So in this work, we studied whether the expression level of *ACE2* was associated with immune infiltration levels in different cancer types. We determined the correlations of *ACE2* levels with immune infiltration levels in 38 cancer types. The data demonstrated that *ACE2* level was significantly negatively associated with tumor purity in seven types (bladder urothelial carcinoma [BLCA], BRCA, BRCA‐basal, BRCA‐luminal, cervical squamous cell carcinoma and endocervical adenocarcinoma [CESC], head and neck squamous cell carcinoma [HNSC], pheochromocytoma and paraganglioma [PCPG]) of cancer, indicating *ACE2* is somehow related to recruiting lymphocytes to the niche of the tumor and significantly associated with B‐cell infiltration levels in seven different cancer types (BRCA, KICH, KIRC, PAAD, PCPG, PRAD, UCEC), whereas negatively associated with B‐cell infiltration in OV (Figure S2).

Moreover, the mRNA expression level of *ACE2* has a correlation with infiltrating levels of CD8+ T cells in 10 different types of cancers (BLCA, BRCA, BRCA‐luminal, KICH, KIRC, LGG, PAAD, PRAD, THCA, UCEC), whereas it demonstrates negative correlation with CD8+ T‐cell infiltration in COAD, glioblastoma multiforme (GBM), OV. The level of *ACE2* is significantly correlated with varying infiltration levels of CD4+ T cells in nine kinds of cancer (BRCA, BRCA‐luminal, CESC, COAD, HNSC, HNSC‐HPV_POS_, PAAD, PRAD, STAD), macrophages in five cancer types (KIRC, KIRP, KICH, SAFC, PRAD), neutrophils in 17 cancer types (BLCA, BRCA, BRCA‐basal, BRCA‐luminal, CESC, HNSC, HNSC‐HPVneg, KIRC, mesothelioma, PAAD, OV, PARD, skin cutaneous melanoma‐primary, READ, uterine carcinosarcoma [UCS], UCEC, uveal melanoma), and dendritic cells (DCs) in 11 types of cancer (BRCA, BLCA, CESC, KICH, KIRC, OV, PAAD, PARD, sarcoma [SARC], UCEC, UCS), whereas it has negative correlation with CD4+ T‐cell infiltration in lung squamous cell carcinoma, with macrophages in six kinds of cancer (CESE, ESCA, GBM, HNSC, HNSC‐HPVneg, SAFC), with neutrophils in COAD and GBM, and dendritic cells in LUCS and SARC (Figure S2).

Because there is correlation of *ACE2* expression level with immune infiltration level in various kinds of cancer, next we assessed the distinct types of cancers in which *ACE2* was correlated with prognosis and immune infiltration.

Tumor purity is a key element that dominates the assessment of immune infiltration levels in clinical malignancy samples by using genomic approaches. So in this study, we chose the cancer types in which *ACE2* levels have demonstrated a significant negative association with tumor purity by using TIMER database and it showed a significant correlation with prognosis.

**FIGURE 4:**
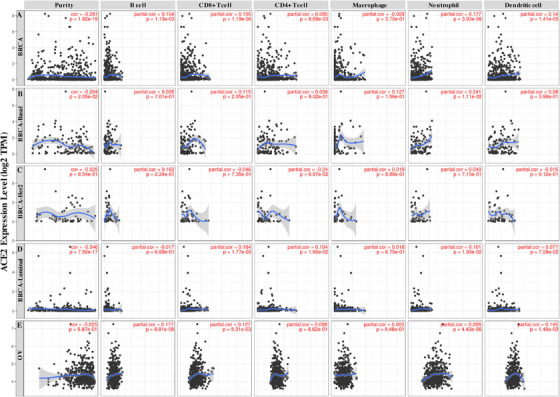
Correlation of *ACE2* expression with immune infiltration level in (A) BRCA, (B) BRCA‐basel, (C) BRCA‐Her2, (D) BRCA‐luminal, and (E) OV. A, *ACE2* expression is significantly negatively related to tumor purity, and has significant positive correlations with infiltrating levels of B cells, CD8+ T cells, CD4+ T cells, neutrophils, and dendritic cells (DCs) in BRCA (n = 1093). B, *ACE2* expression is significantly negatively related to tumor purity, and has significant positive correlations only with the level of neutrophils in BRCA‐basel (n = 139). C, *ACE2* expression is not significantly negatively related to tumor purity, and has no significant correlation with infiltrating level of B cells, CD8+ T cells, CD4+ T cells, macrophages, neutrophils, and DCs in BRCA‐Her2 (n = 67). D, *ACE2* expression is significantly negatively related to tumor purity, and has significant positive correlations with infiltrating levels of CD8+ T cells, CD4+ T cells, and neutrophils in BRCA‐luminal (n = 611). E, *ACE2* expression has no significant correlation with tumor purity, infiltrating level of CD4+ T cells, macrophages, but has significantly positive correlation with infiltrating levels of B cells, CD8+ T cells, neutrophils, and DCs in OV (n = 303).

*What interested us is that there are two different patterns of ACE2 expression associated with OS prognosis and immune infiltration levels in BRCA and OV* (Figure 4). The *ACE2* expression levels of BRCA, BRCA‐basel, and BRCA‐luminal are all significantly negatively related with tumor purity rather than in OV (Figure 4). The expression level of *ACE2* has significant positive associations with infiltration levels of B cells, T cells (CD8+, CD4+), DCs, and neutrophils in BRCA, and only significant positive correlations with infiltration levels of neutrophils in BRCA‐basel, and significant positive correlations with infiltrating levels of T cells (CD8+, CD4+) and neutrophils in BRCA‐luminal, whereas no correlation with any immune infiltration in BRCA‐Her2 (Figure 4). Similarly, there were positive correlations with infiltration levels of B cells, neutrophils, CD8+ T cells, and DCs in OV (Figure 4). More interestingly, the correlation with immune cells demonstrated a different pattern in BRCA and OV. All these above mentioned features strongly imply that *ACE2* plays a crucial role in immune infiltration, maybe CD4+ T cells, in multiple types of cancers, and could lead to a better prognosis in OV patients instead of in BRAC patients.

### 2.5 Correlation between *ACE2* expression level and immune marker sets

**FIGURE 5:**
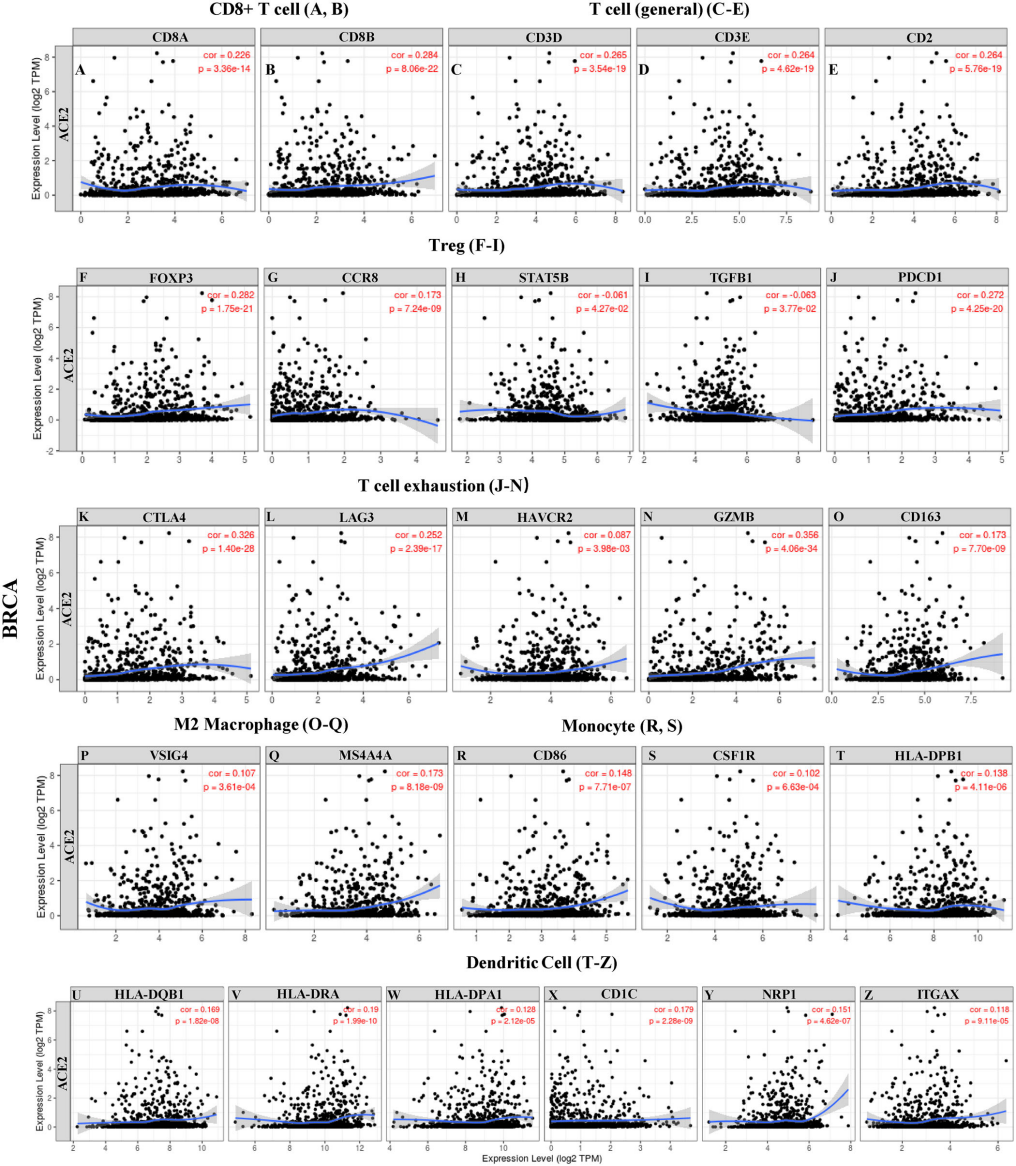
Correlation analysis between *ACE2* expression and immune marker sets in BRCA (n = 1093). Markers include CD8A and CD8B of CD8+ T cell; CD3D, CD3E, and CD2 of general T cell; FOXP3, CCR8, STAT5B, and TGFB1 of Treg; PDCD1, CTLA4, LAG3, HAVCR2, and GZMB of exhausted T cells; CD163, VSIG4, and MS4A4A of M2 macrophages; CD86 and CSF1R of monocytes; HLA‐DPB1, HLA‐DQB1, HLA‐DRA, HLA‐DPA1, CD1C, NRP1, and ITGAX of dendritic cell. Scatterplots of correlations between *ACE2* expression and gene markers of CD8+ T cell (A,B), general T cell (C‐E), Treg (F‐I), T‐cell exhaustion (J‐N), M2 macrophage (O‐Q), monocyte (R,S), and dendritic cell (T‐Z) in BRCA.

**FIGURE 6:**
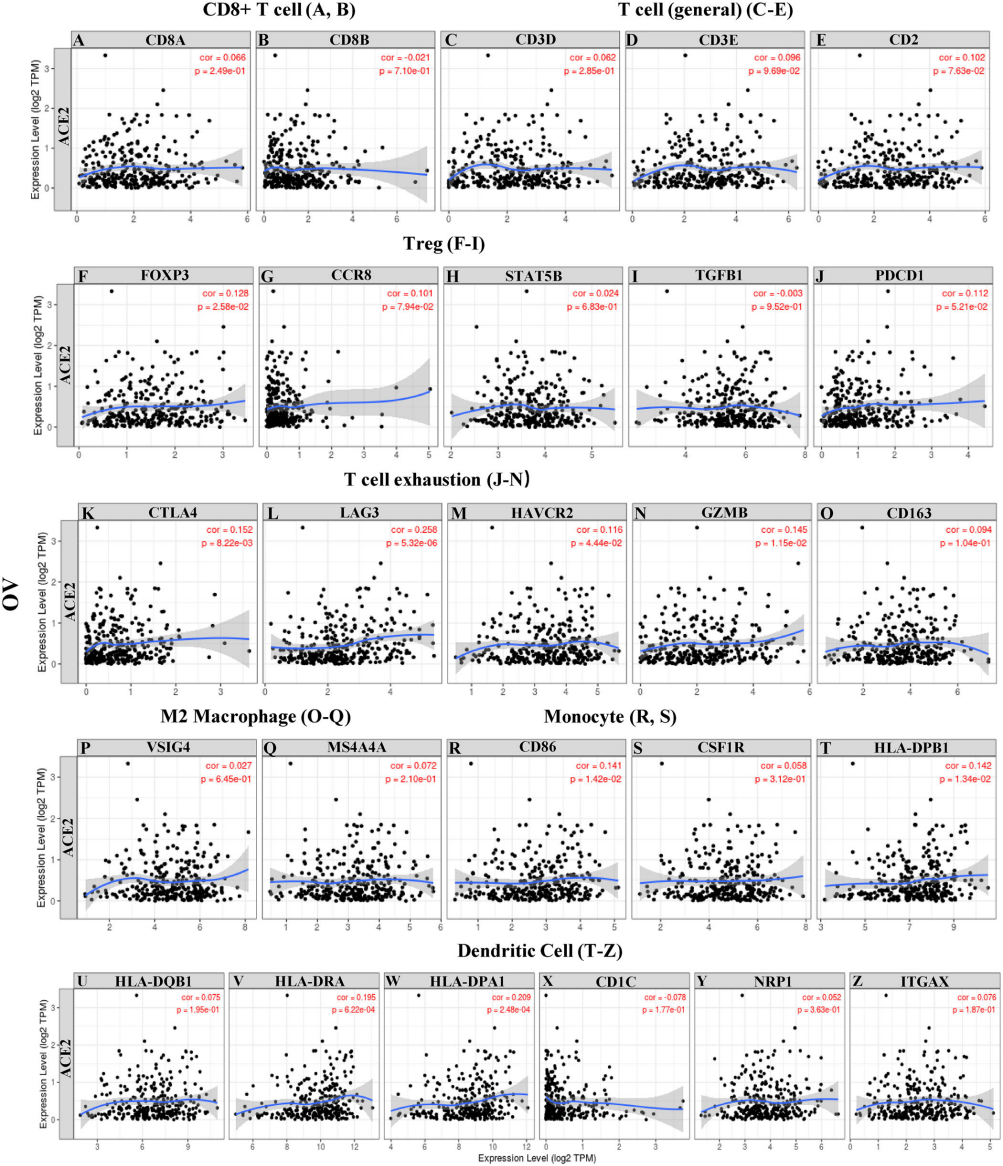
Correlation analysis between *ACE2* expression and immune marker sets in OV (n = 303). Markers include CD8A and CD8B of CD8+ T cell; CD3D, CD3E, and CD2 of general T cell; FOXP3, CCR8, STAT5B, and TGFB1 of Treg; PDCD1, CTLA4, LAG3, HAVCR2, and GZMB of exhausted T cells; CD163, VSIG4, and MS4A4A of M2 macrophages; CD86 and CSF1R of monocytes; HLA‐DPB1, HLA‐DQB1, HLA‐DRA, HLA‐DPA1, CD1C, NRP1, and ITGAX of dendritic cell. Scatterplots of correlations between *ACE2* expression and gene markers of CD8+ T cell (A,B), general T cell (C‐E), Treg (F‐I), T‐cell exhaustion (J‐N), M2 macrophage (O‐Q), monocyte (R,S) and dendritic cell (T‐Z) in OV.

To study the correlation between *ACE2* level and the diverse types of tumor immune‐infiltrating cells (TIICs), we investigated the correlations between *ACE2* and immune marker sets of various immune‐infiltrating cells in BRCA and OV cohorts. We assessed the relationship between the expression level of *ACE2* and the immune marker gene sets of different tumor TIICs, including T (functional and general) cells, B cells, monocytes, tumor‐associated macrophage (TAMs; M1 and M2), NK cells, neutrophils, and DCs (Table S7 and Figure 5). We also investigated the various types of functional T cells, including Th1, Th2, Th17, Tfh, and Tregs, as well as exhausted T cells.^24^, ^25^ After alignment by tumor purity parameter, the consequence demonstrated that the *ACE2* level was significantly associated with most immune marker sets of different immune cells and various subtypes of T cells, especially effect T cells in BRCA cohorts. However, none of these marker set was significantly correlated with *ACE2* level in OV with a better prognosis (Table S7 and Figure 6).

We also discovered that the mRNA expression levels of the marker gene sets in T cells (general, CD8+ T, naive T, effector T), B cells, and natural killer cells demonstrate strong correlations with *ACE2* expression level in BRCA rather than in OV (Table S7). Further investigation needs to be done on whether *ACE2* is the key element that mediates the recruiting immune cells and remodel tumor microenvironment. In addition, *ACE2* also demonstrates correlation with the marker sets of T‐helper cells (including Th1, Th2, Tfh, and Treg cells) in BRCA, but not in OV (Table S7).

### 2.6 *ACE2* mediates different correlation patterns between subtypes in BRCA cohorts

What interests us most is the different correlation patterns among the different subtypes of BRCA patients. There is a significant correlation between *ACE2* level and above‐mentioned immune marker gene sets of various immunocytes, including T cells (general, CD8+ T, Treg, exhausted T), M2 macrophages, monocytes and DCs in *BRCA‐Her2 cohorts*, but none of these above‐demonstrated correlations were there between *ACE2* expression level and the immunocytes’ marker sets in *BRCA‐luminal cohorts* (Figures S3 and S4). There is an intermediate state in BRCA‐basel, that is, significant correlations between *ACE2* level and some of the immune cells mentioned above, including T cells (general, exhausted T), M2 macrophages, monocytes and some genes (HLA‐DQB1, HLA‐DPA1, HLA‐DRA) of DCs (Figure S5).

Furthermore, we investigated the correlation between the mRNA expression level of *ACE2* and the above marker sets of various types of T cells (naïve T, effector T, effector memory T, resident memory T, central memory T, resting Treg, exhausted T, effector Treg, Th1‐like) in BRCA, BRCA‐basel, BRCA‐luminal, and BRCA‐her2. Correlation results between *ACE2* and markers of various types of T cells are similar to the above mentioned (Figures S6‐S9).

## 3 DISCUSSION

This study offers an overview of COVID‐19 infection‐related vulnerable cancer patients. Based on the RNA‐seq and Affymetrix microarray datasets, we stratified cancer patients into high‐ and low‐risk groups according to the mRNA expression levels of *ACE2*. The clinical symptoms of COVID‐19 infection, such as diarrhea, dyspnoea, and kidney failure, have been proved to be related to the invasion of COVID‐19 in upper respiratory track, lung, and kidney, but there is a lack of systematic research on the effect on cancer patients.^26^, ^27^, ^28^, ^29^, ^30^ Here, we designated high and low vulnerable cancer patients based on *ACE2* expression levels. Many studies have shown that the COVID‐19 invasion is not just via *ACE2*, which implies that our work might not exhaust all COVID‐19 infection‐related vulnerable cancer patients. Moreover, as the gene expression may vary among individuals, in‐deepth research is still required to exclude the susceptibility of the cancer patients who were categorized as low risk.

Next, we also reported that variations in mRNA expression level of *ACE2* correlate to prognosis in various kinds of cancers. We found that higher *ACE2* level could lead to a better prognosis in ovarian cancer and poorer prognosis in breast cancer. Furthermore, our finding demonstrates that the immune infiltration levels and multiple immune marker sets are correlated to *ACE2* expression levels in different subtypes of BRCA. We also analyzed the protein level of *ACE2* in these human organs via Human Protein Atlas. The protein expressions of *ACE2* were consistent with our results, supporting the predictive risk map of different organs based on RNA‐seq and microarray data. Our work also provides insights into elucidating the potential role of *ACE2* in tumor immunology and the usage as a cancer biomarker and novel therapeutic target for ovarian cancer and breast cancer. These correlations are the indications of a promising potential mechanism that *ACE2* modulates the function of T cells in breast cancer and ovarian cancer.

All these findings imply that *ACE2* plays an essential role in recruitment and regulation of the effective T cells infiltrating in breast cancer leading to a poorer prognosis. There are also some other COVID‐19‐related receptors reported, including Cathepsin L (*CTSL)* and transmembrane protease serine 2 (*TMPRSS2)*, that also contribute to the infection of COVID‐19.^7^ According to study, SARS‐S engages/employs *TMPRSS2* for S protein priming and the efficiency of *ACE2* usage has been found to be the crucial determinant of SARS‐CoV infection. SARS‐S and SARS‐2‐S share ∼76% amino acid identity, and SARS‐2‐S, similarly to SARS‐S, uses *ACE2* and *TMPRSS2* for host cell entry. *CTSL* and Cathepsin B were also identified as mediators of SARS‐CoV‐2 infection, via a similar mechanism as *TMPRSS2*. Our future work is to analyze the relationship between these factors and the basis of vulnerability of COVID‐19 infection in cancer patients on single‐cell level.

All in all, in this study, we provided the predictive risk map of COVID‐19 infection in various types of cancer patient cohorts, and a possible mechanism that offers explanations that the expression level of *ACE2* associates with immune infiltration, leading to better prognosis in specific types of cancer, especially in ovarian cancer, and poorer prognosis in breast cancer. So the interactions between *ACE2* and the immunocytes in the tumor microenvironment could be potential expression for the predictive risk of COVID‐19‐related infection and a potential mechanism for the correlationship of *ACE2* expression level with immune infiltration level and prognosis in cancer patients.

## 4 MATERIALS AND METHODS

### 4.1 Expression analysis

The *ACE2* expression level was identified in the EBI, including in 158 organisms from 29 experiments, 702 cell lines, development stage, disease and individuals in Homo sapiens samples. The *ACE2* expression level in various types of cancers was assessed in Oncomine database, TIMER, and GEPIA2 database.^31^, ^32^, ^33^ The threshold was given as follows: *P*‐value = .0001, fold change = 1.5, and gene ranking top 10%.

### 4.2 Survival prognosis analysis

The correlationship between *ACE2* level and prognosis in various types of cancers was determined via the database of PrognoScan and GEPIA2.^33^, ^34^ The relationships between the expression level of biomarkers and the patients’ prognosis could be searched and analyzed across a large collection of publicly available cancer datasets. The threshold was the Cox *P*‐value < .05.

Kaplan‐Meier plotter gives the correlation between *ACE2* level and survival, as well as various cancer staging in various cancers was assessed on Kaplan‐Meier plotter, which is capable to analyze the effect of more than 50 000 genes on survival base on over 10 000 cancer samples in more than 20 cancer types.^35^ We also analyzed HR with 95% confidence intervals and log‐rank *P*‐value.

### 4.3 Tumor‐infiltrating analysis

TIMER is a well‐established comprehensive resource for studying immune infiltrates across different types of cancers, which uses a deconvolution statistical approach to surmise the abundance of TIICs from gene expression profiles.^32^ TIMER includes over 10 000 samples from TCGA to estimate the abundance of immune infiltrates. Here, we determined the expression level of *ACE2* across 37 types of cancer and the correlation of the mRNA expression level of *ACE2* with the immune‐infiltrating level, including function T cells (CD4+ and CD8+ T), and B cells, macrophages (M1 and M2), neutrophils, and DCs. Gene expression levels adjusted by tumor purity are also demonstrated.^24^, ^25^ In addition, the correlations between the expression level of *ACE2* and marker sets of TIICs were also explored. The gene markers of TIICs contained the marker sets of various T cells (general T, Th1, Th2, Tfh, Th17, Tregs, exhausted T), B cells, TAMs, monocytes, macrophages (M1 and M2), neutrophils, NK cells, and DCs.^36^, ^37^ This correlation module could generate an expression scatter plot between a pair of user‐defined genes in a specific cancer type, as well as Spearman's correlation and statistical significance. This expression level of the genes was exhibited with log2 RSEM.

### 4.4 Gene correlation analysis

In the next step, we further confirmed the significantly correlated genes via GEPIA2 database, which is a comprehensive resource for gene expression analysis based on nearly 10 000 paired tumor and normal samples from the TCGA and the GTEx databases, to generate overall survival curves and disease‐free survival (DFS) based on the genes expression and the Mantel‐Cox test in various types of cancer. GEPIA2 is an interactive web server featuring 84 cancer subtypes and supports analysis of a specific cancer subtype and comparison between/among subtypes.^33^ Gene expression correlation assessment was implemented for given sets of TCGA expression data.

### 4.5 Statistical analysis

The statistical analysis was as per our previous work. The results produced via Oncomine are exhibited with *P*‐values, fold changes, and ranks. The consequence of Kaplan‐Meier plots, PrognoScan, and GEPIA are exhibited with HR and *P* or Cox *P*‐values from a log‐rank test. The correlation coefficient of gene expression was evaluated by Spearman's correlation and *P‐*values < .05 were considered statistically significant.

## CONFLICT OF INTEREST

The authors declare that there is no conflict of interest.

## ETHICS STATEMENT

This project was permitted by the Independent Ethics Committee of Shanghai Jiao Tong University. This research conforms to all the laws and ethical guidelines that apply in the country.

## AUTHOR CONTRIBUTIONS

Jian He carried out the literature review, analyzed the datasets, and wrote the manuscript. Hui Wang and Xufang Yang reviewed and proof read the article.

## Supporting information

Supporting Information

Supporting Information

Supporting Information

## Data Availability

The data that support the findings of this study are available from the corresponding authors upon request.
