# Supplementary figures and images for: Construction of a risk map to understand the vulnerability of various types of cancer patients to COVID‐19 infection
Source: MedComm (2020). 2021 Jan 21;2(1):69–81. doi: 10.1002/mco2.53 (PMC8014155; doi:10.1002/mco2.53)

**Figure S1**

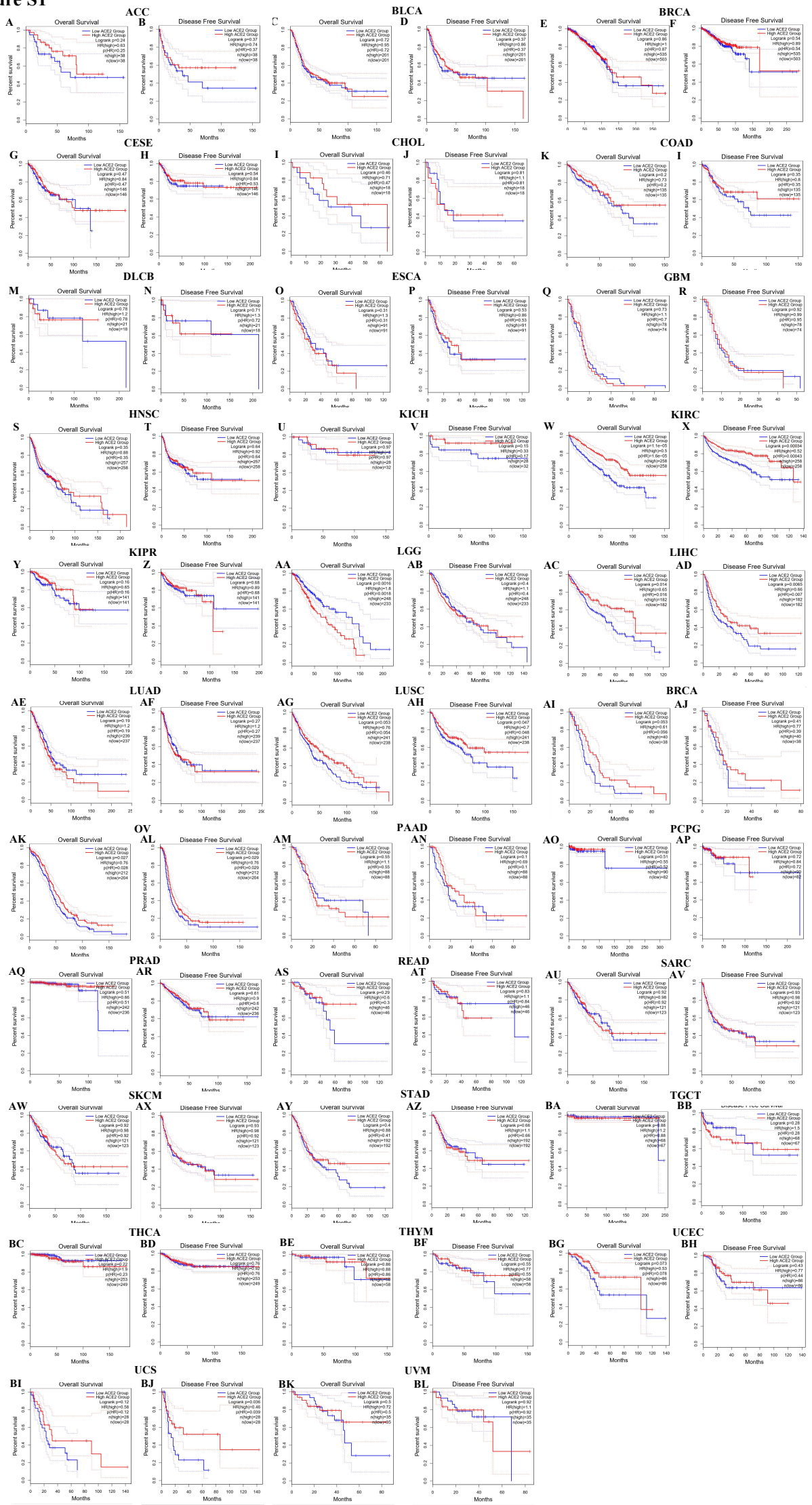

**Figure S2**

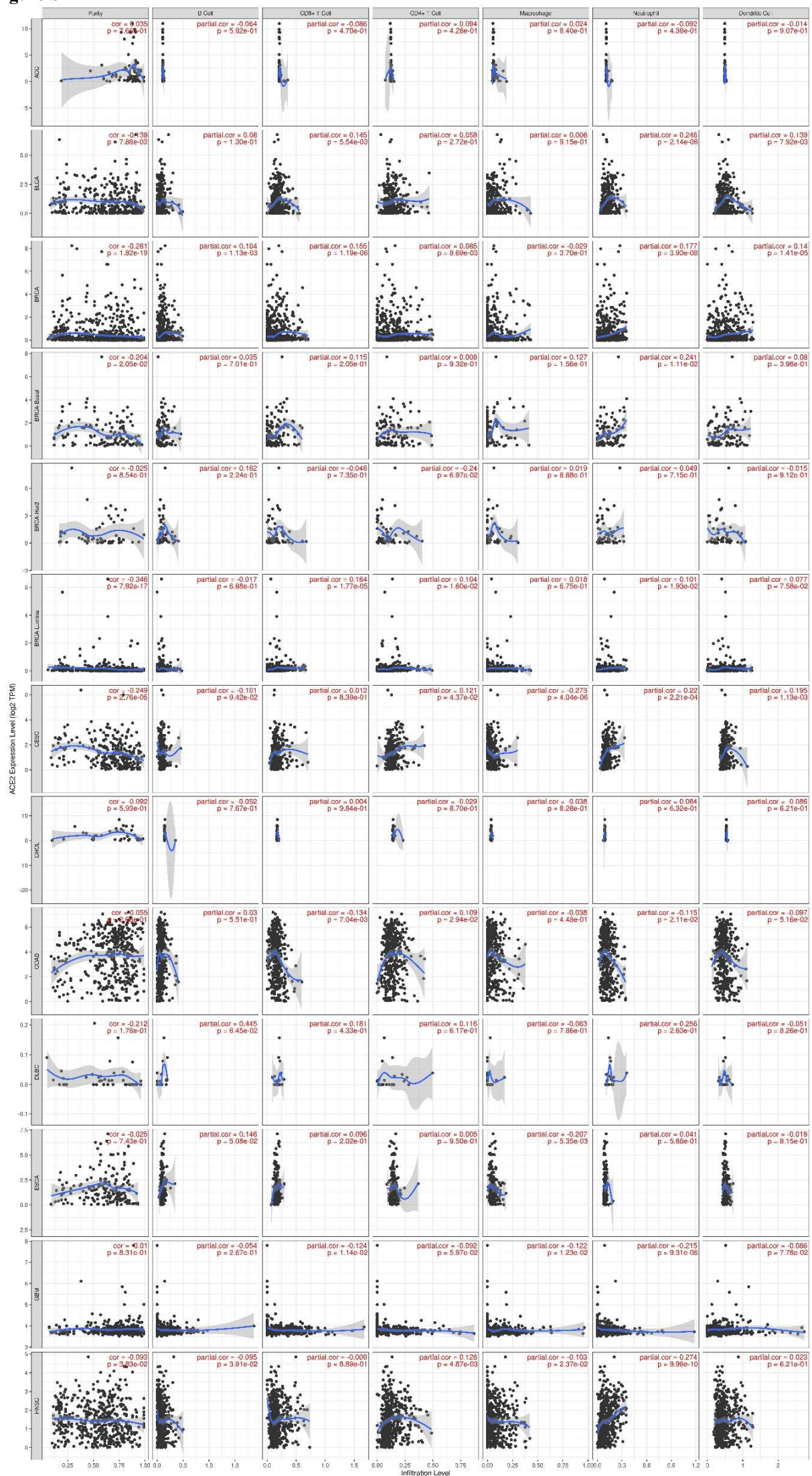

**Figure S2**

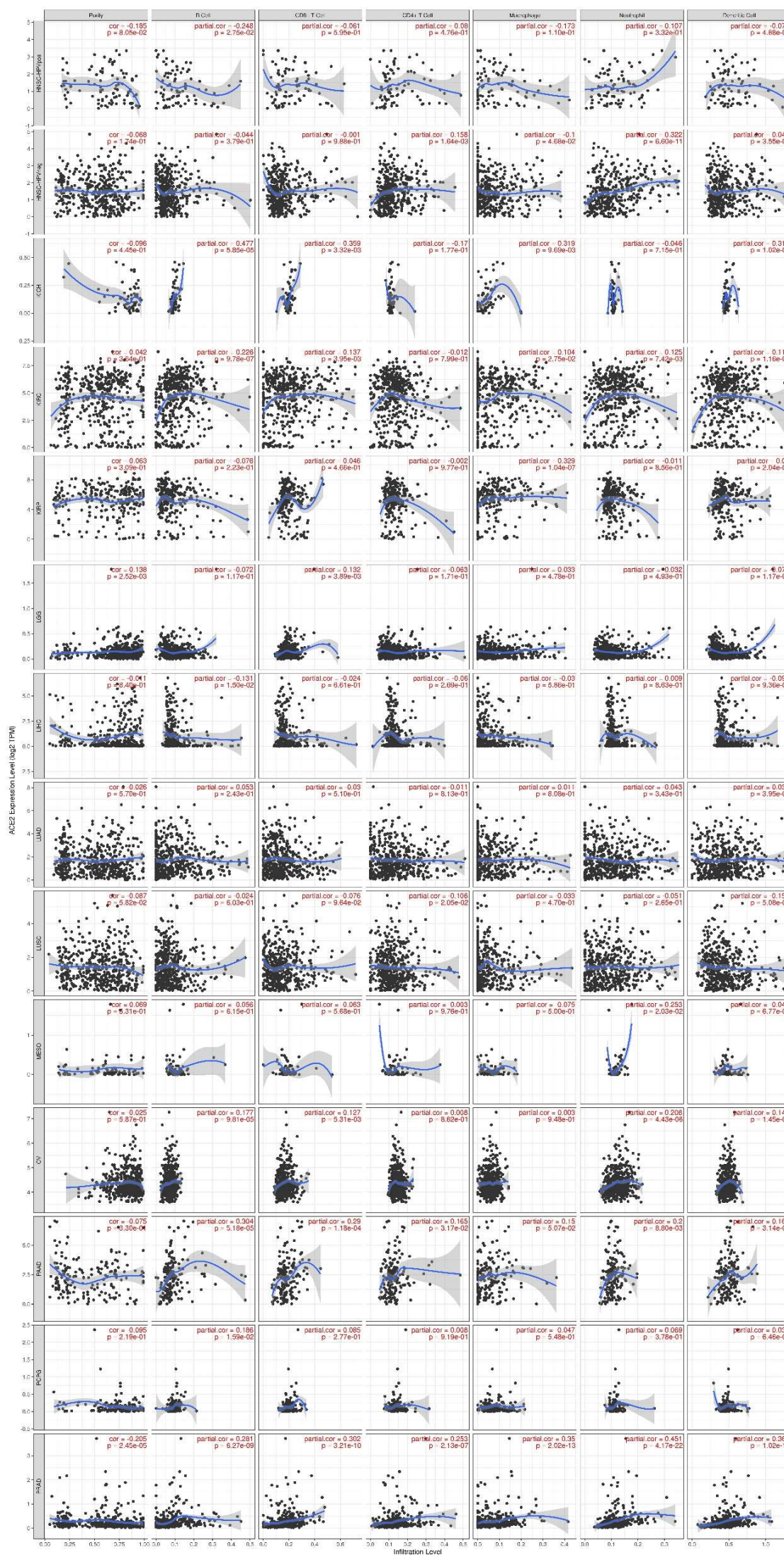

**Figure S2**

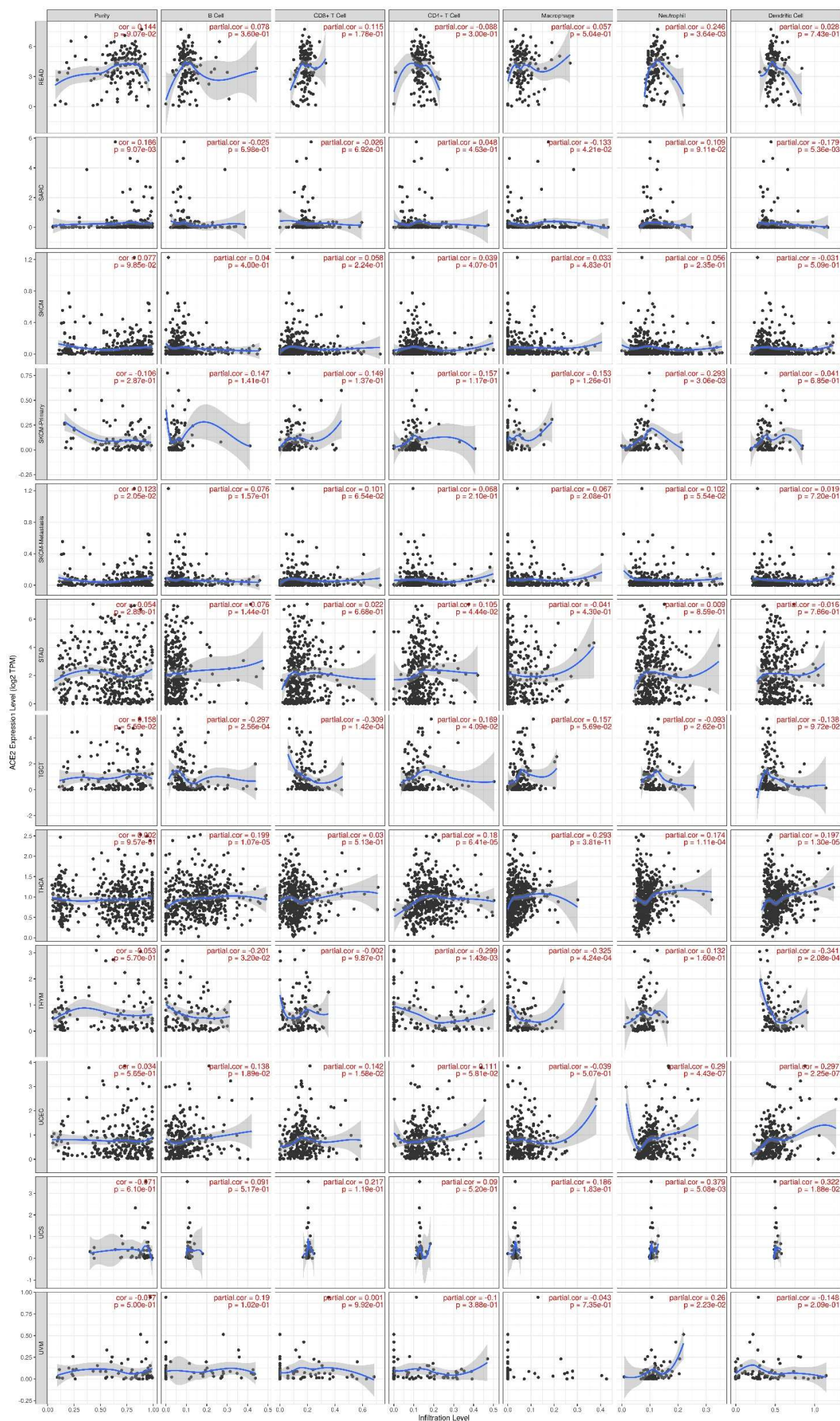

**Figure S3**

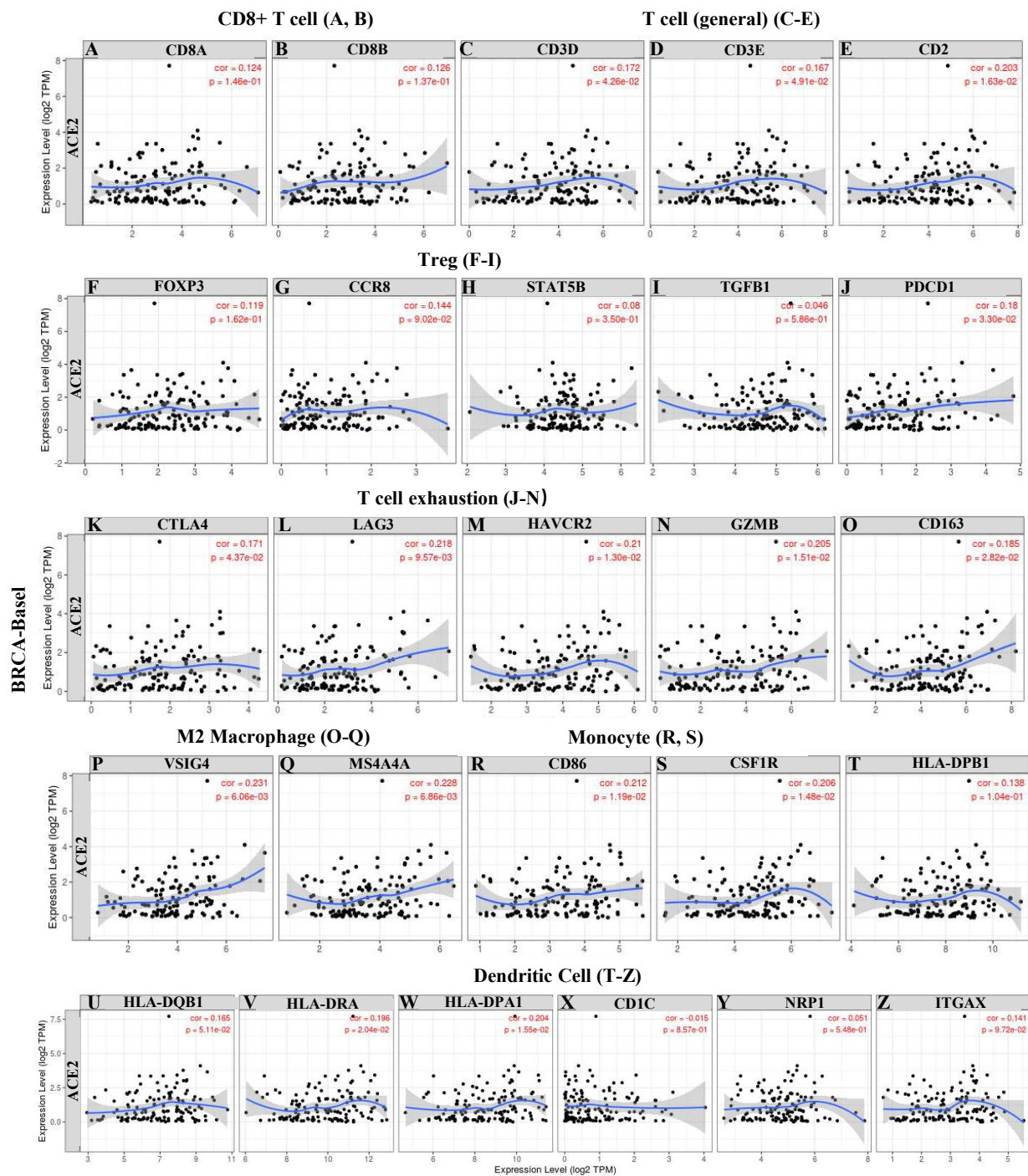

**Figure S4**

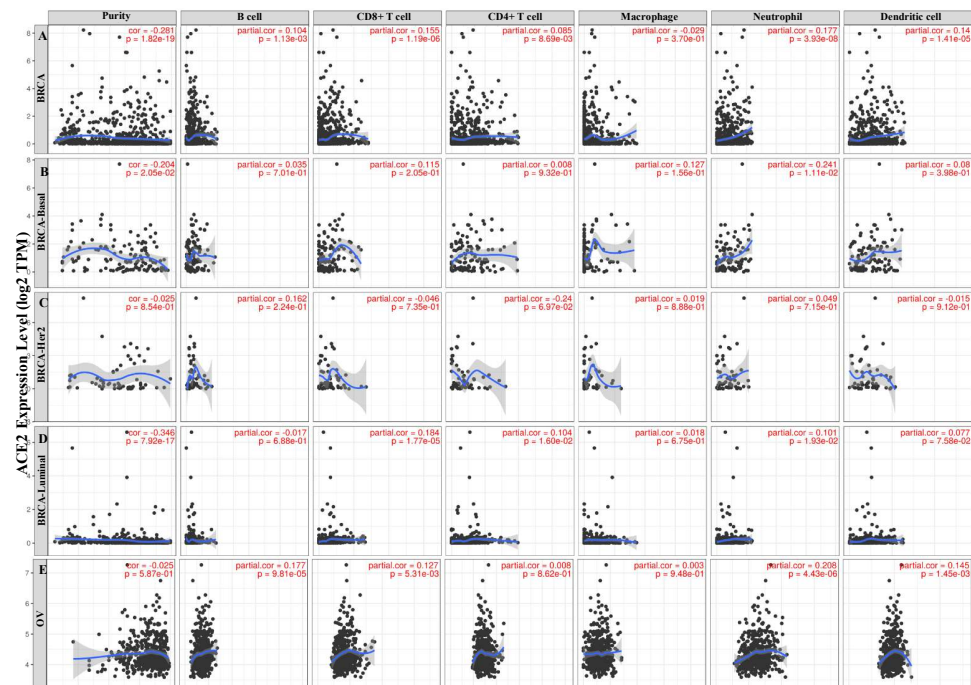

**Figure S5**

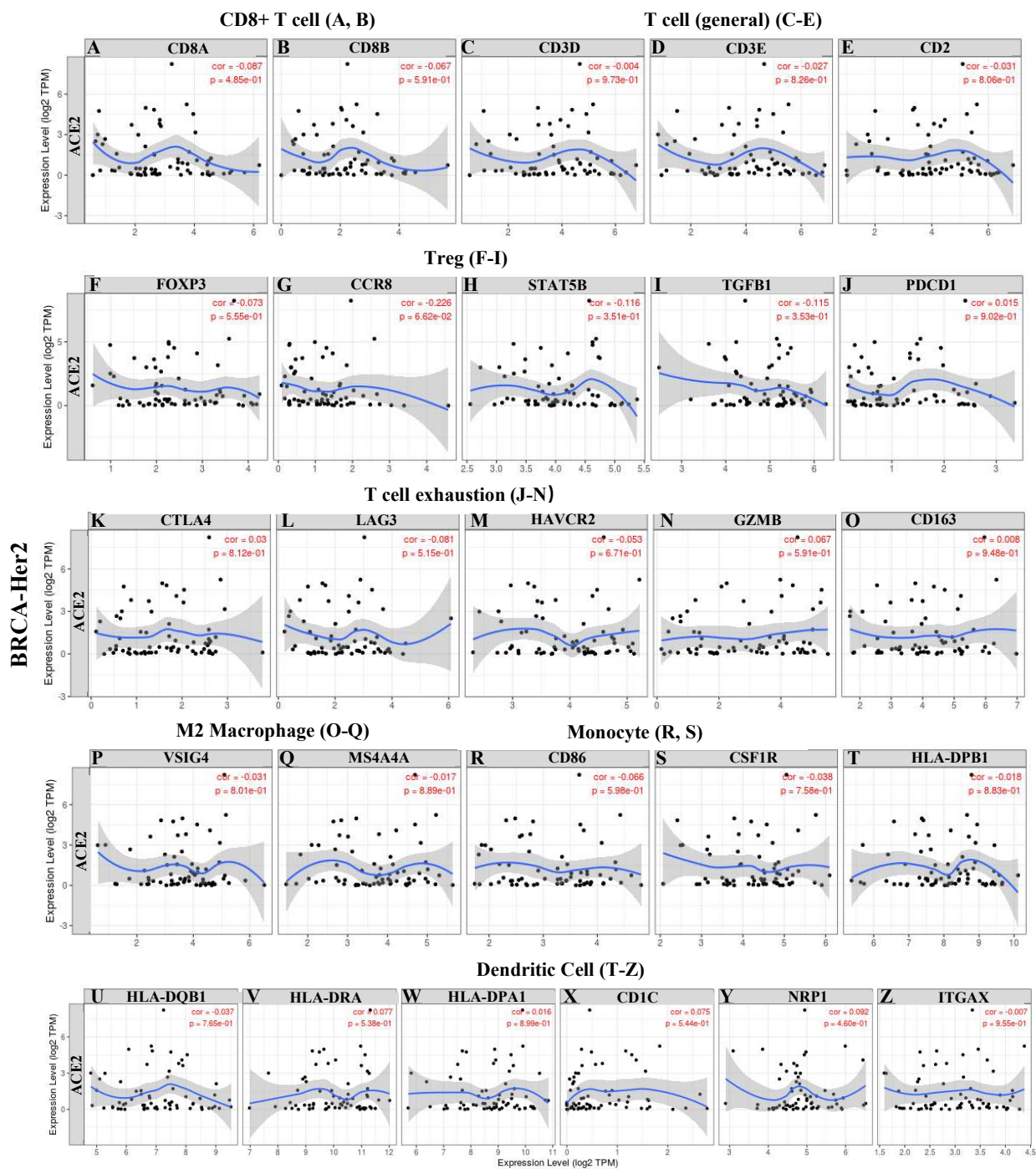

**Figure S6**

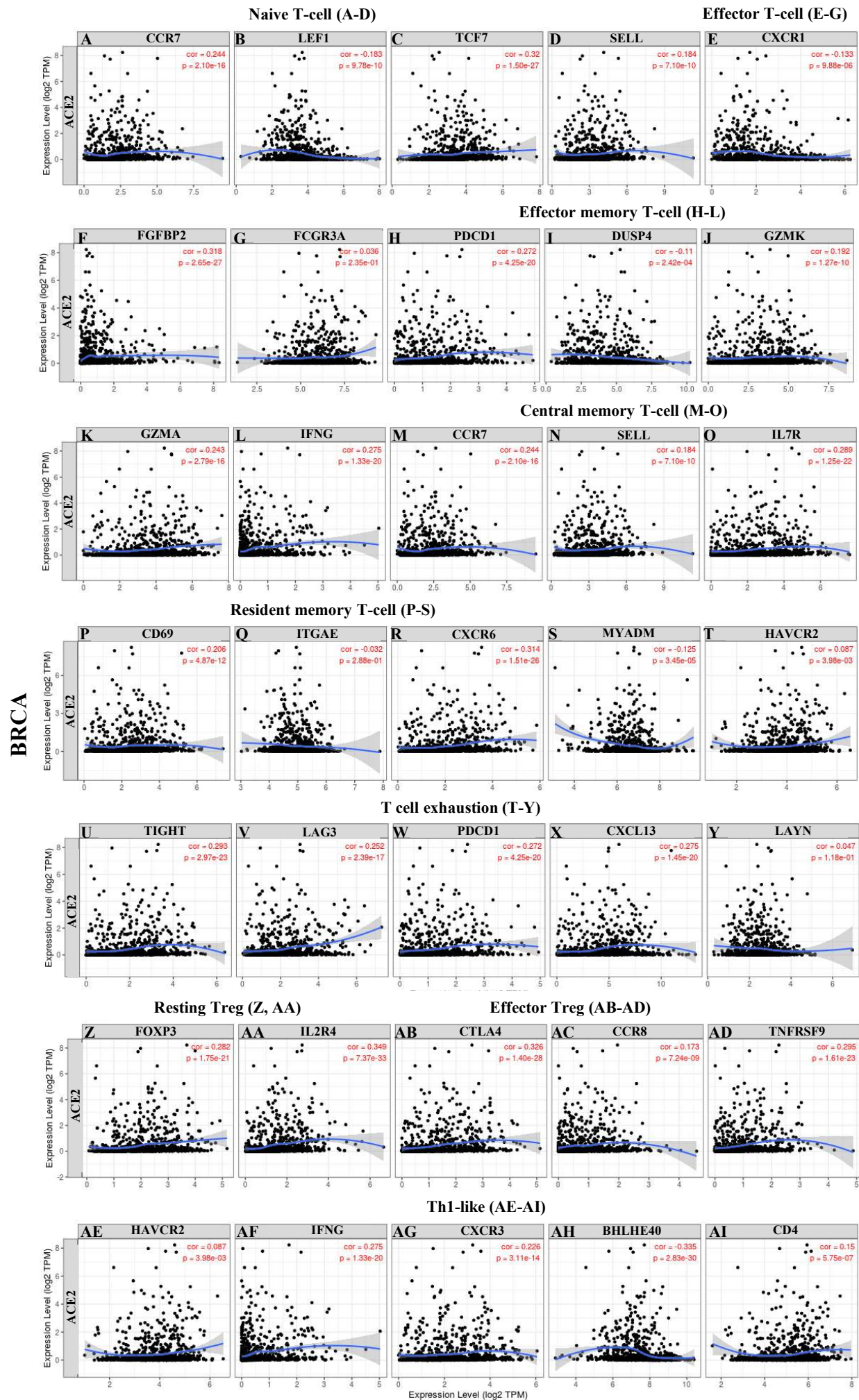

**Figure S7**

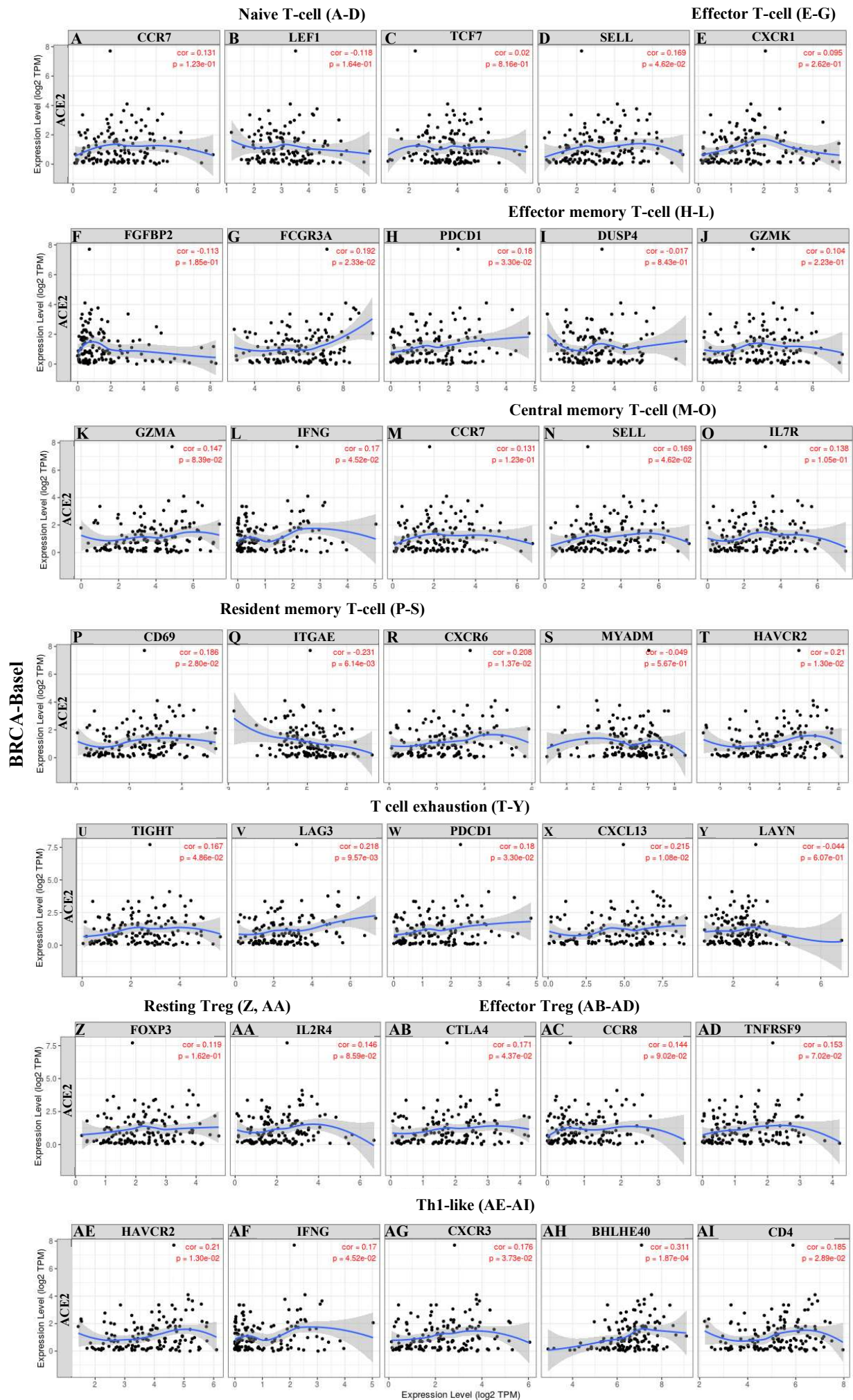

**Figure S8**

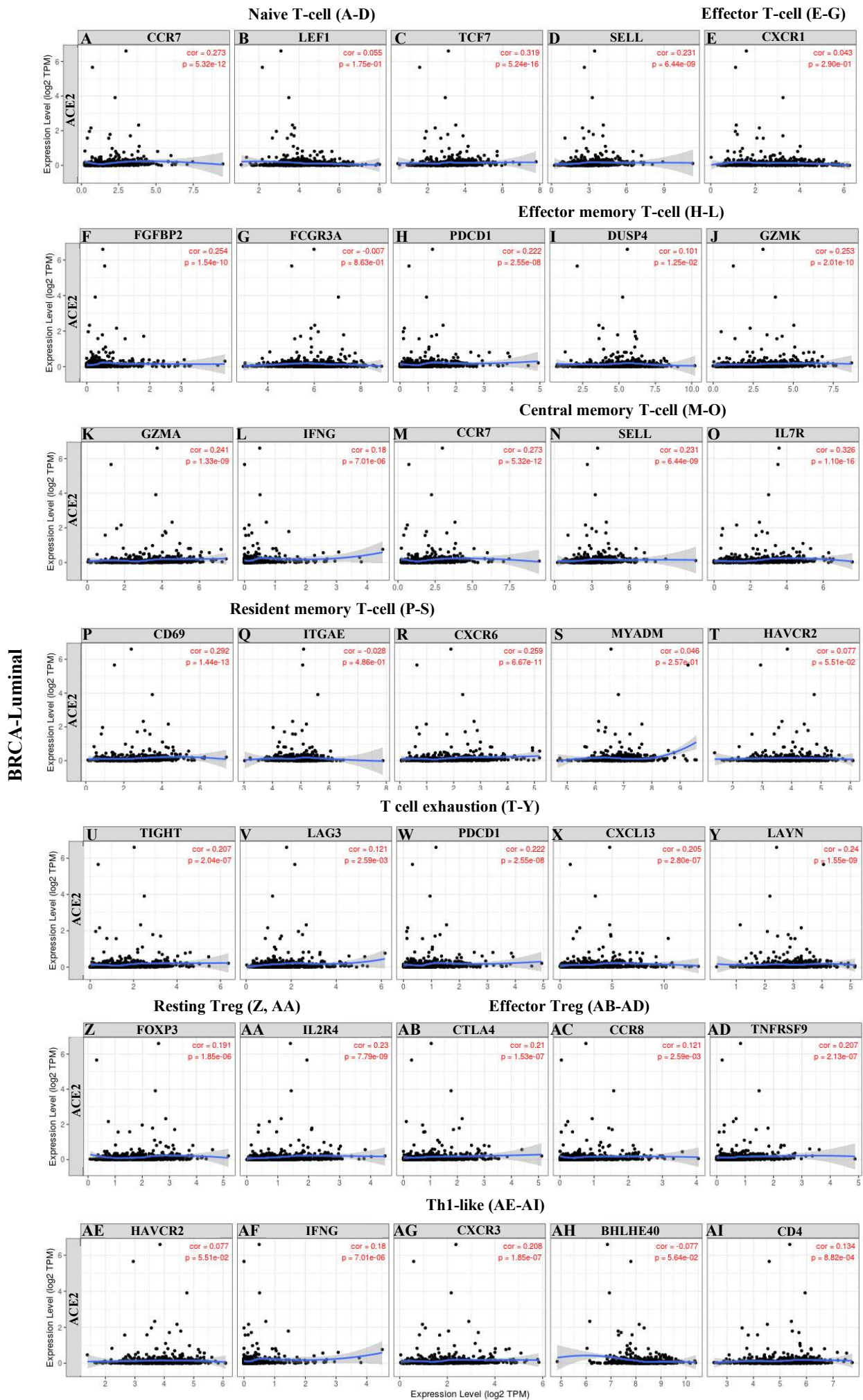

**Figure S9**

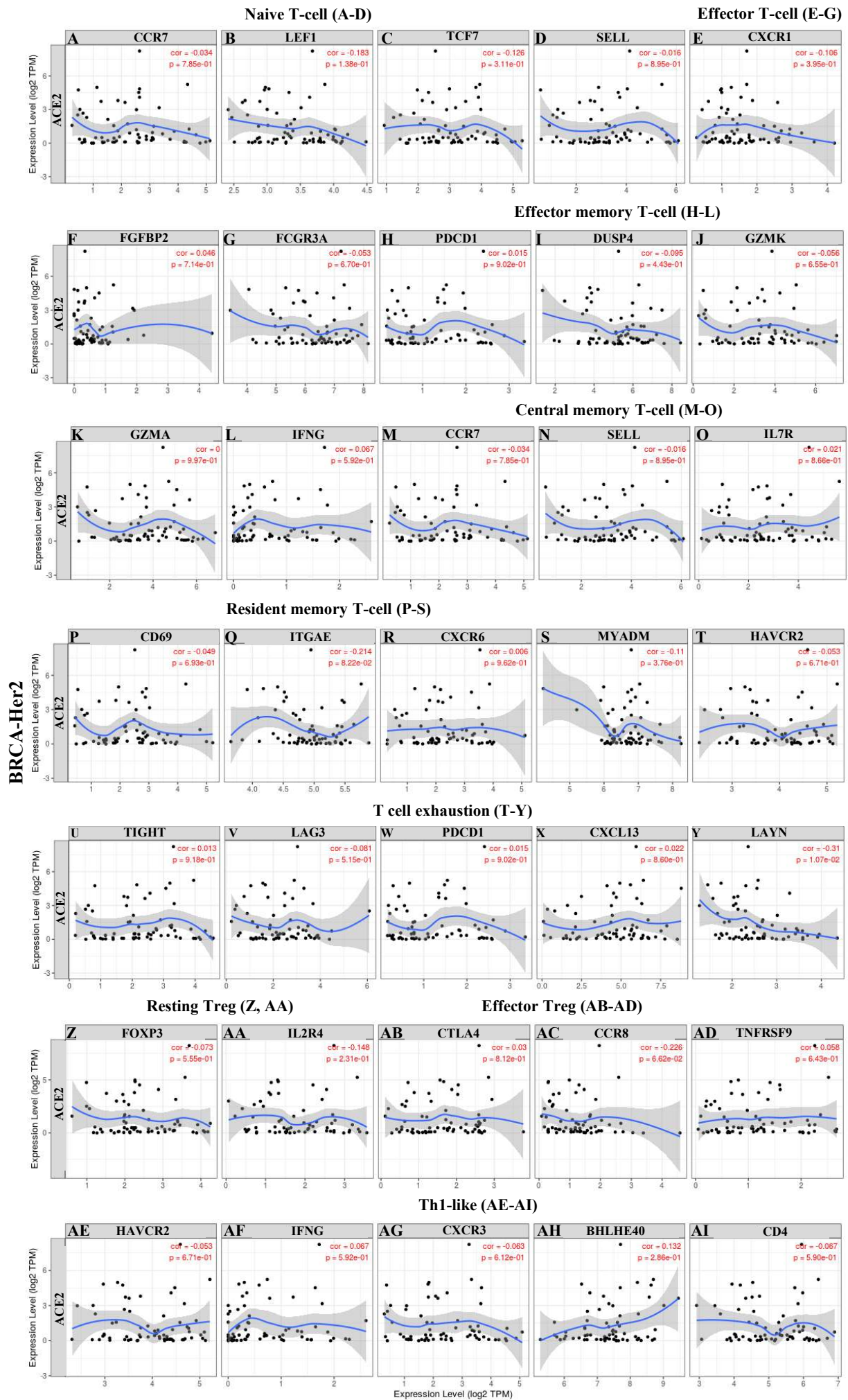

Supplement: Supplementary file 1 — Supporting Information [file MCO2-2-69-s003.pdf]
