# Supplementary material for: Construction of a risk map to understand the vulnerability of various types of cancer patients to COVID‐19 infection
Source: MedComm (2020). 2021 Jan 21;2(1):69–81. doi: 10.1002/mco2.53 (PMC8014155; doi:10.1002/mco2.53)
Supplement: Supplementary file 2 — Supporting Information [file MCO2-2-69-s002.docx]

| **Table S1. *ACE2* expression in Oncomine database** | | | | | | |
| --- | --- | --- | --- | --- | --- | --- |
| **Cancer** | **Cancer type (Cancer vs Normal)** | ***p-value*** | **Fold change** | **Rank (%)** | **Sample** | **PubMed ID** |
| Breast Cancer | Invasive Breast Carcinoma | 2.11E-16 | 2.279 | 4 | 725 | [18438415](https://www.ncbi.nlm.nih.gov/pubmed/18438415) |
|  | Invasive Lobular Breast Carcinoma | 1.93E-11 | -3.314 | 5 | 931 | TCGA |
|  | Invasive Ductal Breast | 9.75E-26 | -2.684 | 6 | 1124 | TCGA |
| Colorectal Cancer | Colon Mucinous Adenocarcinoma | 9.11E-5 | -2.690 | 8 | 1413 | 17615082 |
|  | Colon Adenoma | 8.55E-5 | -3.739 | 9 | 1650 | 20957034 |
| Kidney Cancer | Chromophobe Renal Cell Carcinoma | 1.50E-10 | -5.131 | 2 | 212 | 16115910 |
|  | Renal Pelvis Urothelial Carcinoma | 1.12E-6 | -3.856 | 7 | 841 | 16115910 |
|  | Renal Oncocytoma | 1.17E-7 | -4.528 | 9 | 1026 | 16115910 |
| Liver Cancer | Cirrhosis | 6.51E-9 | 1.755 | 9 | 1054 | 19098997 |
| Lung Cancer | Lung Adenocarcinoma | 1.36E-11 | 2.039 | 5 | 866 | 22080568 |
| Lymphoma | Germinal Center B-Cell-Like Diffuse Large B-Cell Lymphoma | 2.94E-11 | -1.872 | 1 | 118 | 19412164 |
|  | Activated B-Cell-Like Diffuse Large B-Cell Lymphoma | 1.06E-10 | -1.841 | 3 | 556 | 19412164 |
|  | Follicular Lymphoma | 1.81E-9 | -1.649 | 9 | 1568 | 19412164 |
|  | Diffuse Large B-Cell Lymphoma | 2.15E-10 | -1.809 | 10 | 1800 | 19412164 |
| Other Cancer | Yolk Sac Tumor, NOS | 1.64E-5 | -2.621 | 9 | 1710 | 17767167 |
|  | Seminoma, NOS | 3.01E-12 | -14.554 | 1 | 46 | 16424014 |
|  | Mixed Germ Cell Tumor, NOS | 1.48E-19 | -12.891 | 1 | 50 | 16424014 |
|  | Embryonal Carcinoma, NOS | 2.54E-10 | -13.188 | 2 | 282 | 16424014 |
| Sarcoma | Gastrointestinal Stromal Tumor | 8.51E-5 | -3.067 | 7 | 1335 | 21447720 |

| Table S3. Correlation of *ACE2* expression and prognosis in ovarian cancer with different clinicopathological factors (222257_s_at) | | | | | | |
| --- | --- | --- | --- | --- | --- | --- |
| **Clinicopathological Characteristics** | Overall survival (n =1656) | | | Progression-free survival (n = 1435) | | |
|  | N | Hazard ratio | *p-value* | N | Hazard ratio | *p-value* |
| **Histology** | | | | | | |
| Endometrioid | 37 | 0.39 (0.04 – 3.46) | 0.38 | 51 | 0.33 (0.11 – 1.03) | **0.045** |
| Serous | 1207 | 0.85 (0.73 – 0.99) | **0.039** | 1104 | 0.92 (0.78 – 1.09) | 0.34 |
| **Stage** | | | | | | |
| 1 | 74 | 0.2 (0.06 – 0.62) | **0.002** | 96 | 2.32 (0.77 – 6.98) | 0.12 |
| 1+2 | 135 | 0.17 (0.08 – 0.39) | **1.7E-06** | 163 | 0.34 (0.19 – 0.63) | **0.00031** |
| 2 | 61 | 0.18 (0.06 – 0.56) | **0.00099** | 67 | 0.55 (0.26 – 1.13) | 0.1 |
| 2+3 | 1105 | 0.83 (0.7 – 0.99) | **0.037** | 986 | 1.12 (0.97 – 1.31) | 0.12 |
| 2+3+4 | 1281 | 0.82 (0.7 – 0.97) | **0.017** | 1148 | 1.09 (0.95 – 1.25) | 0.22 |
| 3 | 1044 | 0.85 (0.72 – 1.02) | 0.078 | 919 | 1.18 (1.02 – 1.38) | **0.03** |
| 3+4 | 1220 | 0.84 (0.72 – 0.99) | **0.041** | 1081 | 1.15 (1 - 1.32) | 0.055 |
| 4 | 176 | 0.7 (0.47 – 1.05) | 0.086 | 163 | 0.85 (0.58 – 1.24) | 0.39 |
| **Grade** | | | | | | |
| 1 | 56 | 1.84 (0.53 – 6.42) | 0.33 | 37 | 4.36 (0.57 – 33.56) | 0.12 |
| 1+2 | 380 | 0.85 (0.63 – 1.14) | 0.28 | 293 | 0.84 (0.63 – 1.11) | 0.2162 |
| 2 | 324 | 0.8 (0.59 – 1.09) | 0.16 | 256 | 0.82 (0.61 – 1.09) | 0.17 |
| 2+3 | 1339 | 0.8 (0.7 – 0.93) | **0.0031** | 1093 | 0.87 (0.74 – 1.03) | 0.11 |
| 3 | 1015 | 0.76 (0.65 – 0.9) | **0.0016** | 837 | 0.88 (0.72 – 1.06) | 0.18 |
| 4 | 20 | -- |  | 19 | -- |  |
| **TP53 mutation** | | | | | | |
| Mutated (n=516) | 506 | 0.9 (0.72 – 1.13) | 0.37 | 483 | 1.46 (1.16 – 1.84) | **0.0011** |
| Wild type (n=102) | 94 | 1.85 (1.04 – 3.27) | **0.032** | 84 | 0.67 (0.39 – 1.16) | 0.15 |

*Bold values indicate p* < *0.05.*

| **Table S4.** Correlation of *ACE2* mRNA expression and clinical prognosis in breast cancer with different clinicopathological factors by Kaplan-Meier plotter (222257_s_at) | | | |
| --- | --- | --- | --- |
| **Clinicopathological Characteristics** | Overall survival (n = 1402) | | |
|  | N | Hazard ratio | *p-value* |
| **ER status (n=3779)** | | | |
| Positive | 548 | 1 (0.69 - 1.43) | 0.99 |
| Negative | 251 | 0.92 (0.58 – 1.46) | 0.73 |
| **PR status (n=1982)** | | | |
| Positive | 83 | 0.88 (0.23 – 3.32) | 0.85 |
| Negative | 89 | 1.11 (0.44 – 2.79) | 0.83 |
| **HER2 status (n=1872)** | | | |
| Positive | 129 | 1.08 (0.53 – 2.2) | 0.8316 |
| Negative | 130 | 0.7 (0.28 – 1.77) | 0.45 |
| **Intrinsic subtype (n=5143)** | | | |
| Basal | 241 | 1.15 (0.7 – 1.88) | 0.5845 |
| Luminal A | 611 | 1.2 (0.85 – 1.71) | 0.3 |
| Luminal B | 433 | 0.74 (0.51 – 1.09) | 0.1295 |
| HER2+ | 117 | 0.62 (0.32 – 1.2) | 0.15 |
| **Lymph node status (n=3720)** | | | |
| Lymph node positive | 313 | 0.84 (0.57 – 1.25) | 0.3938 |
| Lymph node negative | 594 | 1.02 (0.7 – 1.48) | 0.93 |
| **Grade (n=2545)** | | | |
| 1 | 161 | 1.19 (0.46 – 3.08) | 0.7226 |
| 2 | 387 | 1.04 (0.68 – 1.61) | 0.8532 |
| 3 | 503 | 0.83 (0.6 – 1.15) | 0.2645 |
| **TP53 status (n=595)** | | | |
| Mutated | 111 | 1.19 (0.55 – 2.6) | 0.6539 |
| Wild type | 187 | 0.76 (0.4 – 1.45) | 0.4037 |
| **Pietenpol subtype (n=1246)** | | | |
| Basal-like 1 | 58 | 0.82 (0.27 – 2.44) | 0.7192 |
| Basal-like 2 | 38 | 2.03 (0.57 – 7.28) | 0.2679 |
| Immunomodulatory | 100 | 0.9 (0.35 – 2.26) | 0.8161 |
| Mesenchymal | 73 | 0.98 (0.44 – 2.16) | 0.9515 |
| Mesenchymal stem-like | 19 | -- | -- |
| Luminal androgen receptor | 83 | 0.73 (0.37 – 1.45) | 0.36 |

| Table S5. Correlation of *ACE2* mRNA expression and clinical prognosis in ovarian cancer with different treatments by Kaplan-Meier plotter (222257_s_at) | | | | | | | |
| --- | --- | --- | --- | --- | --- | --- | --- |
| **Treatments** | Overall survival (n =1656) | | | | Progression-free survival (n = 1435) | | |
|  | N | | Hazard ratio | *p-value* | N | Hazard ratio | *p-value* |
| **Debulk** | | | | | | | |
| Optimal | 801 | 0.75 (0.59 – 0.97) | | **0.027** | 696 | 0.84 (0.68 – 1.03) | 0.093 |
| Suboptimal | 536 | 0.79 (0.63 – 0.98) | | **0.0307** | 459 | 0.84 (0.67 – 1.06) | 0.14 |
| **Chemotherapy** | | | | | | | |
| Contains platin | 1409 | 0.78 (0.66 – 0.92) | | **0.0033** | 1259 | 0.87 (0.75 – 1.01) | 0.06 |
| Contains Taxol | 793 | 0.74 (0.59 – 0.92) | | **0.0076** | 715 | 0.89 (0.73 – 1.08) | 0.25 |
| Contains Taxol + platin | 776 | 0.73 (0.58 – 0.91) | | **0.005** | 698 | 0.89 (0.73 – 1.09) | 0.26 |
| Contains Avastin | 50 | 2.37 (0.91 - 6.16) | | 0.069 | 50 | 0.42 (0.19 – 0.92) | 0.025 |
| Contains Docetaxel | 108 | 1.71 (0.99 – 2.98) | | 0.054 | 106 | 0.59 (0.34 – 1.02) | 0.057 |
| Contains Gemcitabine | 135 | 1.31 (0.87 – 1.97) | | 0.19 | 131 | 1.42 (0.97 – 2.09) | 0.073 |
| Contains Paclitaxel | 220 | 0.54 (0.31 – 0.94) | | **0.026** | 229 | 0.78 (0.56 – 1.1) | 0.16 |
| Contains Topotecan | 119 | 1.69 (1.11 – 2.57) | | **0.014** | 118 | 1.8 (1.21 – 2.68) | 0.0035 |

*Bold values indicate p* < *0.05.*

| Table S6. Correlation of *ACE2* mRNA expression and clinical prognosis in breast cancer patients with following systemic treatment by Kaplan-Meier plotter (222257_s_at) | | | |
| --- | --- | --- | --- |
| **Treatments** | Overall survival (n = 1402) | | |
|  | N | Hazard ratio | *P-value* |
| **Endocrine therapy** | | | |
| Include | 215 | 0.83 (0.48 – 1.42) | 0.4906 |
| Exclude | 631 | 1.21 (0.86 – 1.7) | 0.2835 |
| Tamoxifen only | 146 | 0.65 (0.32 – 1.34) | 0.24 |
| **Chemotherapy** | | | |
| Include all | 300 | 0.98 (0.6 – 1.6) | 0.95 |
| Adjuvant only | 163 | 0.73 (0.4 – 1.34) | 0.31 |
| Neoadjuvant only | 156 | 0.93 (0.44 – 1.98) | 0.8509 |
| Exclude all | 549 | 1.05 (0.73 – 1.49) | 0.81 |

| Table S7. Correlation analysis between *ACE2* and relate genes and markers of immune cells | | | | | | | | | |
| --- | --- | --- | --- | --- | --- | --- | --- | --- | --- |
| **Description** | **Gene markers** | **BRCA (n=1093)** | | | | **OV (n=303)** | | | |
|  |  | None | | Adjusted by  Tumor Purity | | None | | Adjusted by  Tumor Purity | |
|  |  | Cor | *p* | Cor | *p* | Cor | *p* | Cor | *p* |
| **CD8+ T cell** | CD8A | 0.226 | *** | 0.116 | *** | 0.066 | 0.249 | 0.069 | 0.277 |
|  | CD8B | 0.284 | *** | 0.191 | *** | -0.021 | 0.71 | -0.049 | 0.443 |
| **T Cell (general)** | CD3D | 0.265 | *** | 0.146 | *** | 0.062 | 0.285 | 0.047 | 0.456 |
|  | CD3E | 0.264 | *** | 0.145 | *** | 0.096 | 0.0969 | 0.101 | 0.111 |
|  | CD2 | 0.264 | *** | 0.154 | *** | 0.102 | 0.0763 | 0.107 | 0.0912 |
| **Naive T-Cell** | CCR7 | 0.244 | *** | 0.128 | *** | 0.087 | 0.131 | 0.086 | 0.177 |
|  | LEF1 | -0.183 | *** | -0.267 | *** | 0.053 | 0.357 | 0.086 | 0.174 |
|  | TCF7 | 0.32 | *** | 0.231 | *** | 0.046 | 0.427 | 0.051 | 0.427 |
|  | SELL | 0.184 | *** | 0.058 | 0.067 | 0.255 | *** | 0.305 | *** |
| **Effector T-Cell** | CX3CR1 | -0.133 | *** | -0.214 | *** | 0.067 | 0.245 | 0.02 | 0.749 |
|  | FGFBP2 | 0.318 | *** | 0.227 | *** | -0.139 | 0.0152 | -0.146 | 0.0214 |
|  | FCGR3A | 0.036 | 0.235 | -0.016 | 0.623 | 0.112 | 0.0521 | 0.147 | 0.0204 |
| **Effector memory T-Cell** | PDCD1 | 0.272 | *** | 0.172 | *** | 0.112 | 0.0521 | 0.146 | 0.0214 |
|  | DUSP4 | -0.11 | ** | -0.141 | *** | -0.035 | 0.548 | 0.022 | 0.727 |
|  | GZMK | 0.192 | *** | 0.057 | 0.0736 | -0.034 | 0.554 | -0.072 | 0.258 |
|  | GZMA | 0.243 | *** | 0.131 | *** | 0.076 | 0.186 | 0.094 | 0.141 |
|  | IFNG | 0.275 | *** | 0.196 | *** | 0.109 | 0.0578 | 0.109 | 0.0849 |
| **Resident memory T-Cell** | CD69 | 0.206 | * | 0.074 | * | 0.161 | * | 0.17 | ** |
|  | ITGAE | -0.032 | 0.288 | -0.015 | 0.647 | -0.118 | 0.0399 | -0.107 | 0.0916 |
|  | CXCR6 | 0.314 | *** | 0.222 | *** | 0.127 | 0.0267 | 0.156 | 0.0139 |
|  | MYADM | -0.125 | *** | -0.141 | *** | 0.068 | 0.235 | 0.049 | 0.44 |
| **B cell** | CD19 | 0.273 | *** | 0.156 | *** | 0.043 | 0.457 | 0.031 | 0.624 |
|  | CD79A | 0.283 | *** | 0.163 | *** | -0.053 | 0.358 | -0.095 | 0.134 |
| **Monocyte** | CD86 | 0.148 | *** | 0.06 | 0.0569 | 0.141 | 0,0142 | 0.174 | * |
|  | CD115 (CSF1R) | 0.102 | ** | -0.016 | 0.618 | 0.058 | 0.312 | 0.071 | 0.266 |
| **TAM** | CCL2 | 0.293 | *** | 0.228 | *** | 0.01 | 0.863 | 0.016 | 0.805 |
|  | CD68 | 0.093 | * | 0.006 | 0.842 | 0.098 | 0.0877 | 0.119 | 0.0605 |
|  | IL10 | 0.166 | *** | 0.096 | *** | 0.017 | 0.767 | 0.003 | 0.963 |
| **M1 Macrophage** | INOS  (NOS2) | 0.09 | * | 0.063 | 0.0454 | 0.08 | 0.165 | 0.127 | 0.0461 |
|  | IRF5 | 0.09 | * | -0.048 | 0.133 | 0.209 | ** | 0.221 | ** |
|  | COX2 (PTGS2) | 0.402 | *** | 0.331 | *** | -0.005 | 0.931 | -0.006 | 0.923 |
| **M2 Macrophage** | CD163 | 0.173 | *** | 0.11 | ** | 0.094 | 0.104 | 0.116 | 0.0683 |
|  | VSIG4 | 0.107 | ** | 0.032 | 0.314 | 0.027 | 0.645 | 0.055 | 0.384 |
|  | MS4A4A | 0.173 | *** | 0.086 | * | 0.072 | 0.21 | 0.111 | 0.0804 |
| **Neutrophils** | CD66b (CEACAM8) | 0.069 | 0.0217 | 0.074 | 0.0202 | 0.028 | 0.625 | 0.03 | 0.637 |
|  | CD11b (ITGAM) | 0.066 | 0.0294 | -0.021 | 0.503 | 0.067 | 0.244 | 0.059 | 0.357 |
|  | CCR7 | 0.244 | *** | 0.128 | *** | 0.087 | 0.131 | 0.086 | 0.177 |
| **Natural killer cell** | KIR2DL1 | 0.165 | *** | 0.112 | ** | 0.088 | 0.128 | 0.089 | 0.161 |
|  | KIR2DL3 | 0.188 | *** | 0.115 | ** | 0.166 | * | 0.174 | * |
|  | KIR2DL4 | 0.273 | *** | 0.207 | *** | 0.307 | *** | 0.358 | *** |
|  | KIR3DL1 | 0.188 | *** | 0.114 | ** | 0.105 | 0.067 | 0.129 | 0.0413 |
|  | KIR3DL2 | 0.234 | *** | 0.15 | *** | 0.062 | 0.279 | 0.062 | 0.333 |
|  | KIR3DL3 | 0.125 | *** | 0.092 | * | 0.133 | 0.207 | 0.123 | 0.052 |
|  | KIR2DS4 | 0.219 | *** | 0.151 | *** | 0.061 | 0.288 | 0.07 | 0.274 |
| **Dendritic cell** | HLA-DPB1 | 0.138 | *** | -0.017 | 0.589 | 0.142 | 0.0134 | 0.184 | * |
|  | HLA-DQB1 | 0.169 | *** | 0.071 | 0.0247 | 0.075 | 0.195 | 0.062 | 0.333 |
|  | HLA-DRA | 0.19 | *** | 0.068 | 0.0318 | 0.195 | ** | 0.234 | ** |
|  | HLA-DPA1 | 0.128 | *** | -0.007 | 0.824 | 0.209 | ** | 0.254 | *** |
|  | BDCA-1 (CD1C) | 0.179 | *** | 0.032 | 0.313 | -0.078 | 0.177 | -0.131 | 0.0393 |
|  | BDCA-4 (NRP1) | 0.151 | *** | 0.078 | 0.0137 | 0.052 | 0.363 | 0.075 | 0.235 |
|  | CD11c (ITGAX) | 0.118 | *** | 0.003 | 0.935 | 0.076 | 0.187 | 0.063 | 0.324 |
| **Th1** | TBX21  (T-bet) | 0.285 | *** | 0.185 | *** | 0.142 | 0.0133 | 0.161 | 0.0109 |
|  | STAT4 | 0.303 | *** | 0.201 | *** | 0.149 | * | 0.14 | 0.0267 |
|  | STAT1 | 0.154 | *** | 0.127 | *** | 0.388 | *** | 0.431 | *** |
|  | IFNG  (IFN-g) | 0.275 | *** | 0.196 | *** | 0.109 | 0.0578 | 0.109 | 0.0849 |
|  | TNF-a (TNF) | 0.172 | *** | 0.123 | *** | -0.014 | 0.806 | -0.032 | 0.614 |
| **Th2** | GATA3 | -0.553 | *** | -0.522 | *** | 0.032 | 0.582 | 0.032 | 0.611 |
|  | STAT6 | -0.112 | ** | -0.151 | *** | 0.206 | ** | 0.193 | * |
|  | STAT5A | 0.149 | *** | 0.082 | * | 0.079 | 0.168 | 0.039 | 0.54 |
|  | IL13 | 0.212 | *** | 0.16 | *** | 0 | 0.994 | 0.011 | 0.859 |
| **Tfh** | BCL6 | 0.076 | 0.012 | 0.027 | 0.396 | 0.07 | 0.225 | 0.105 | 0.099 |
|  | IL21 | 0.202 | *** | 0.15 | *** | 0.08 | 0.167 | 0.038 | 0.554 |
| **Th17** | STAT3 | 0.036 | 0.237 | 0.017 | 0.601 | 0.096 | 0.0946 | 0.113 | 0.0759 |
|  | IL17A | 0.194 | *** | 0.158 | *** | 0.051 | 0.381 | 0.08 | 0.206 |
| **Treg** | FOXP3 | 0.282 | *** | 0.187 | *** | 0.128 | 0.0258 | 0.145 | 0.0221 |
|  | CCR8 | 0.173 | *** | 0.109 | ** | 0.101 | 0.0794 | 0.081 | 0.205 |
|  | STAT5B | -0.061 | 0.0427 | -0.093 | ** | 0.024 | 0.683 | 0.004 | 0.952 |
|  | TGFB1 (TGFb) | -0.063 | 0.0377 | -0.196 | *** | -0.003 | 0.952 | 0.001 | 0.99 |
| **T cell exhaustion** | PDCD1  (PD-1) | 0.272 | *** | 0.172 | *** | 0.112 | 0.0521 | 0.146 | 0.0214 |
|  | CTLA4 | 0.326 | *** | 0.244 | *** | 0.152 | * | 0.194 | * |
|  | LAG3 | 0.252 | *** | 0.201 | *** | 0.258 | *** | 0.311 | *** |
|  | HAVCR2  (TIM-3) | 0.087 | * | 0.002 | 0.939 | 0.116 | 0.444 | 0.143 | 0.0238 |
|  | GZMB | 0.356 | *** | 0.281 | *** | 0.145 | 0.0115 | 0.211 | ** |

*TAM, tumor-associated macrophage; Th, T helper cell; Tfh, Follicular helper T cell; Treg, regulatory T cell;*

*Cor, R value of Spearman’s correlation; None, correlation without adjustment. Purity, correlation adjusted by purity.*

**P* < *0.01;* ***P* < *0.001;* ****P* < *0.0001.*
